# Co‐Delivery of Multiple Toll‐Like Receptor Agonists and Avian Influenza Hemagglutinin on Protein Nanoparticles Enhances Vaccine Immunogenicity and Efficacy

**DOI:** 10.1002/adhm.202404335

**Published:** 2025-02-09

**Authors:** Aaron Ramirez, Jenny E. Hernandez‐Davies, Aarti Jain, Lu Wang, Erwin Strahsburger, D. Huw Davies, Szu‐Wen Wang

**Affiliations:** ^1^ Department of Chemical and Biomolecular Engineering University of California Irvine CA 92697 USA; ^2^ Vaccine Research and Development Center Department of Physiology and Biophysics University of California Irvine CA 92697 USA; ^3^ Institute for Immunology University of California Irvine CA 92697 USA; ^4^ Department of Biomedical Engineering University of California Irvine CA 92697 USA; ^5^ Chao Family Comprehensive Cancer Center University of California Irvine CA 92697 USA

**Keywords:** CpG, flagellin, immunomodulation, influenza vaccine, protein nanoparticle

## Abstract

Most seasonal and pandemic influenza vaccines are derived from inactivated or attenuated virus propagated in chicken eggs, while more advanced delivery technologies, such as the use of recombinant proteins and adjuvants, are under‐utilized. In this study, the E2 protein nanoparticle (NP) platform is engineered to synthesize vaccines that simultaneously co‐deliver influenza hemagglutinin (H5) antigen, TLR5 agonist flagellin (FliCc), and TLR9 agonist CpG 1826 (CpG) all on one particle (termed H5‐FliCc‐CpG‐E2), with uniform molecular orientation significant for immunomodulation. Antigen‐bound NP formulations elicit higher IgG antibody responses and broader homosubtypic cross‐reactivity against different H5 variants than unconjugated antigen alone. IgG1/IgG2c skewing is modulated by adjuvant type and NP attachment. Conjugation of flagellin to the NP causes significant IgG1 (Th2) skewing while attachment of CpG yields significant IgG2c (Th1) skewing, and simultaneous conjugation of both flagellin and CpG results in a balanced IgG1/IgG2c (Th2/Th1) response. Animals immunized with E2‐based NP vaccines and subsequently challenged with H5N1 influenza show 100% survival, and only animals that receive adjuvanted NP formulations are also protected against morbidity. This investigation highlights that NP‐based delivery of antigen and multiple adjuvants can be designed to effectively modulate the strength, breadth toward variants, and bias of an immune response against influenza viruses.

## Graphical abstract

This research highlights a novel application of protein nanoparticles to effectively modulate the strength, breadth, and bias of an immune response against influenza virus. Nanoparticles precisely co‐delivering antigen (H5) and multiple adjuvants (CpG and FliCc) present a way to improve and direct prophylactic immune responses which can be applicable as a general vaccine approach.

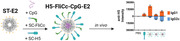

## 1 Introduction

Subunit vaccines such as recombinant protein vaccines have been shown to be safer than attenuated or inactivated vaccines. However, these recombinant proteins tend to suffer from weaker immunogenicity caused by rapid draining kinetics, reduced immunostimulatory adjuvant capacity, and variant pharmacokinetics of subunit vaccine components.^[^ ^1^, ^2^ ^]^ For this reason, the inclusion of pattern recognition receptor (PRR) agonists, such as pathogen associated molecular patterns (PAMPs), as immunoenhancing components is explored with some success, but these molecules suffer from similar issues of differential and reduced pharmacokinetics when administered as soluble adjuvants.^[^ ^3^ ^]^

Nanoparticle (NP)‐based delivery of vaccine antigen and adjuvant components has shown to be a promising solution by combining the safety and tunability of subunit vaccines with enhanced immunogenicity.^[^ ^4^, ^5^, ^6^ ^]^ The increased size of NPs relative to soluble antigen or adjuvant and the ability to repetitively display antigen or adjuvant give NP platforms intrinsic advantages over conventional subunit vaccines. Studies have shown that dendritic cells preferably take up NPs between 20 and 500 nm, with an optimal uptake of ≈20–50 nm.^[^ ^2^, ^7^, ^8^, ^9^ ^]^ NPs in this size range have also been shown to exhibit longer retention times within draining lymph nodes.^[^ ^2^, ^9^, ^10^ ^]^ Investigations have also described size and antigen display topography to play a crucial role in B cell engagement; NPs between 20 and 50 nm in diameter with antigen valences greater than 5 have been reported to more effectively engage B cell receptor cross‐linking and activation.^[^ ^2^ ^]^ These size and antigen orientation effects collectively increase antigen‐specific responses toward NP‐based vaccines.

In light of this, many NP‐based vaccines build upon a “pathogen‐mimetic strategy” of achieving sizes and structure comparable to that of viral or bacterial pathogens and displaying both protein antigens and PAMP molecules. Our previous studies using the E2 protein NP platform have shown its capability to co‐deliver single endosomal toll‐like receptor (TLR) agonists with target antigens within a ∼25–45 nm particle size, for the development of cancer and infectious disease vaccines.^[^ ^11^, ^12^, ^13^ ^]^ However, the addition of a second, different TLR agonist to the same protein NP scaffold has yet to be explored, despite previous studies that have shown that combinations of agonists for endosomal‐based (TLR3, 7, 8, 9) and cell‐surface‐based (TLR1, 2, 4, 5, 6) TLRs in vaccine formulations can elicit improved immune responses.^[^ ^14^, ^15^, ^16^ ^]^ For that reason, in this study, we engineer protein NPs capable of co‐delivering endosomal and cell‐surface TLR agonists with antigen on a single NP and investigate the prophylactic immune responses elicited by these NPs.

To our knowledge, no study to date has used a NP to co‐deliver two different TLR agonists and a target antigen conjugated on the same vaccine particle with consistent and uniform molecular orientation. For example, in the context of nanoparticulate structures, multiple TLR agonists have been co‐delivered with protein/peptide antigens, but these have often been in emulsion‐based formulations^[^ ^17^, ^18^, ^19^, ^20^ ^]^ or PLGA or lipid nanoparticles,^[^ ^21^, ^22^, ^23^, ^24^ ^]^ the syntheses of which do not allow for the consistent antigen surface display or orientation that is favorable for B cell receptor engagement. Furthermore, in emulsion‐based formulations, adjuvants and antigens in the soluble phase of the emulsion are not attached to each other, so diffusion and simultaneous transport of individual components to immune cells after injection is not well‐controlled. Others have examined protein‐based NPs to co‐deliver TLR agonists with antigen; this strategy improves molecular orientation, but these investigations have been limited to only one TLR agonist used^[^ ^25^, ^26^, ^27^, ^28^ ^]^ or two TLR agonists but only one was conjugated to the NP.^[^ ^29^, ^30^ ^]^

To build upon this body of work and test the advantages of multiple‐component co‐delivery on NPs, we have engineered E2 protein NPs that can conjugate and deliver two TLR agonists (flagellin [a TLR5 agonist] and CpG [a TLR9 agonist]) and influenza H5 antigen on a single NP, attached in a way that displays each component in its native‐pathogenic orientation. The most well‐studied flagellin is derived from *Salmonella typhimurium*, which does not require glycosylation for adjuvant activity and has been expressed and purified from *Escherichia coli*.^[^ ^31^, ^32^, ^33^, ^34^ ^]^ Flagellin has been shown to elicit Th1 and Th2 immune responses,^[^ ^35^, ^36^, ^37^ ^]^ and combining flagellin into H1 influenza vaccine formulations enhances antibody and T cell responses.^[^ ^35^, ^36^, ^38^ ^]^ In the majority of studies, flagellin is solubly co‐administered with antigen; however, an alternative strategy genetically incorporates flagellin with a recombinant virus expressing influenza antigen.^[^ ^35^, ^36^, ^37^ ^]^ The Th1‐skewing TLR9 agonist, CpG 1826, is the second adjuvant conjugated to our current E2 NP vaccine formulations in this study. Our group has previously synthesized CpG‐loaded E2 protein NPs and demonstrated their efficient dendritic cell activation capability in vitro, as well as potent CD8+ T cell responses in tumor vaccination models.^[^ ^11^, ^39^, ^40^, ^41^ ^]^ In this current study, this protein scaffold is engineered to encapsulate molecules in its hollow interior (e.g., CpG) and display guest proteins on its surface (e.g., flagellin, antigen).^[^ ^13^, ^42^ ^]^

In recent years, the World Health Organization has acknowledged the growing pandemic risk of avian influenza (H5N1), with its zoonotic infection raising concerns that humans will have very little immunity against the virus and its subsequent mutated variants.^[^ ^43^, ^44^ ^]^ For this reason, the immunodominant influenza hemagglutinin (HA) protein antigen (subtype H5 A/Vietnam/1194/2004) (H5) was utilized as the model antigen to investigate in this vaccine strategy. Due to the protein nature of flagellin and HA, both can be recombinantly engineered with the SpyTag/SpyCatcher bioconjugation system, allowing for their separate expression and subsequent conjugation to the E2 nanoparticle via spontaneous isopeptide bond formation.^[^ ^13^, ^45^, ^46^, ^47^, ^48^ ^]^ In this work, we synthesized NPs displaying flagellin, CpG, and HA, investigated the bioactivity of flagellin when displayed on a NP in vitro, evaluated the antibodies elicited after immunization (for strength, breadth toward different H5 variants, and bias of immune response), and demonstrated the protection from viral challenge elicited by adjuvanted NPs. Development of a modular vaccine platform capable of improving and directing prophylactic immune responses by the precise delivery of multiple adjuvants and antigen on a NP could prove extremely favorable as a general vaccine approach.

## 2 Results and Discussion

### 2.1 Conjugation of TLR Agonists Flagellin (FliC) and CpG 1826 (CpG) and Antigen (H5) Onto a Single E2 Nanoparticle

#### 2.1.1 Design of NPs and Their Individual Adjuvant and Antigen Components

**Figure 1:**
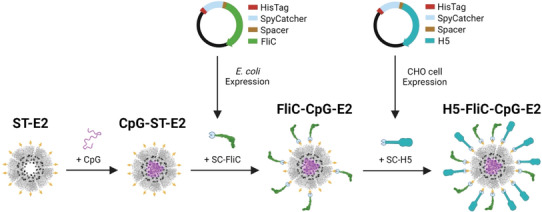
Schematic of nanoparticle synthesis with two adjuvants, CpG and flagellin (FliC), and the immunodominant influenza antigen hemagglutinin (H5). CpG is first conjugated into the internal cavity of the SpyTag‐E2 (ST‐E2) NP via hydrazone bond formation. SpyCatcher‐fused flagellin (SC‐FliC) is then attached to the surface of CpG‐ST‐E2 NPs via ST/SC bond formation. Lastly, SpyCatcher‐fused H5 hemagglutinin (SC‐H5) is attached to the surface of FliC‐CpG‐E2 NPs via the remaining available SpyTags on the E2 NP surface.

The SpyTag(ST)/SpyCatcher(SC) bioconjugation system has become a powerful protein–protein conjugation tool in the fields of vaccines and biomaterials.^[^ ^46^, ^47^, ^48^ ^]^ Here, we apply this system to conjugate the TLR5 agonist flagellin (FliC) and immunodominant influenza antigen hemagglutinin (H5), to the external surface of the E2 protein nanoparticle (**Figure** **1**). Two forms of flagellin are investigated: wild‐type flagellin (FliC) and cysteine‐modified flagellin (FliCc).^[^ ^32^ ^]^ Wild‐type flagellin can be expressed in bacterial expression systems but suffers from C‐terminus degradation.^[^ ^32^ ^]^ The cysteine‐modified flagellin mutant, FliCc, was previously designed to increase the stability of flagellin in solution via internal cross‐links, but it demonstrated 5–10× lower TLR5‐specific activity than FliC. Displaying the cysteine‐modified flagellin on a NP improved its bioactivity in vitro but required the implementation of non‐natural amino acids to allow for a click‐chemistry‐based conjugation. In our studies, we aim to apply an alternative chemical conjugation strategy for displaying flagellin and to examine the resulting bioactivity in vitro and in vivo.

**Figure 2:**
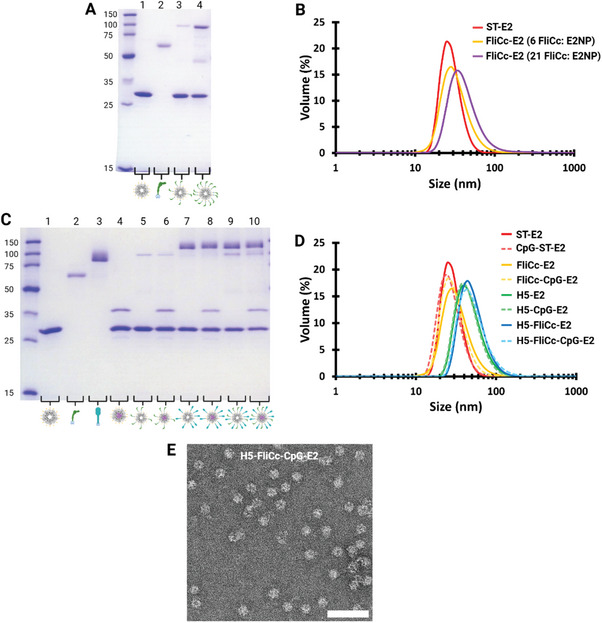
Synthesis of ST‐E2 nanoparticles conjugated with CpG, SC‐FliCc, or SC‐H5. A) SDS‐PAGE showing nanoparticles with ∼6 and ∼21 SC‐FliCc loaded on the external surface. Lanes: 1. ST‐E2; 2. SC‐FliCc; 3. FliCc‐E2 (6 FliCc: E2NP); 4. FliCc‐E2 (21 FliCc: E2NP). MW_ST‐E2_ = 30.2 kDa, MW_SC‐FliCc_ = 65.2 kDa, MW_FliCc‐E2_ = 95.4 kDa. B) Hydrodynamic diameters of FliCc‐E2 NPs with ∼6 and ∼21 SC‐FliCc per nanoparticle conjugated to the external surface. C) SDS‐PAGE of the H5‐FliCc‐CpG‐E2 nanoparticle vaccine showing conjugation of CpG, SC‐FliCc, and SC‐H5 individually and combined on a single nanoparticle. Lanes: 1. ST‐E2; 2. SC‐FliCc; 3. SC‐H5; 4. CpG‐ST‐E2; 5. FliCc‐E2; 6. FliCc‐CpG‐E2; 7. H5‐E2; 8. H5‐CpG‐E2; 9. H5‐FliCc‐E2; and 10. H5‐FliCc‐CpG‐E2. MW_ST‐E2_ = 30.2 kDa, MW_SC‐FliCc_ = 65.2 kDa, MW_SC‐H5_ = 71.4 kDa, MW_CpG_ = 6.6 kDa, MW_FliCc‐E2_ = 95.4 kDa, MW_H5‐E2_ = 101.6 kDa, MW_CpG‐ST‐E2_ = 36.8 kDa, MW_FliCc‐CpG‐E2_ = 102 kDa, and MW_H5‐CpG‐E2_ = 108.2 kDa. SC‐H5 protein migrates from ∼80–90 kDa on SDS‐PAGE due to glycosylation. SC‐H5 conjugates runs ∼10–20 kDa higher than expected on SDS‐PAGE due to glycosylation. D) Hydrodynamic diameters of H5‐E2 NPs showing physical stability and monodispersity of nanoparticles and shifts in size upon conjugation with adjuvants. E) Representative TEM image of the H5‐FliCc‐CpG‐E2 nanoparticles. Scale bar = 100 nm.

The E2 NP with 60 STs displayed on its external surface and 60 internal‐cavity cysteines for CpG attachment (ST‐E2) was utilized in this synthesis strategy.^[^ ^13^ ^]^ SpyCatcher was genetically fused to the N‐terminus of the flagellin and H5 proteins. This ensured that when conjugated to the ST‐E2 nanoparticle, flagellin and H5 would be oriented in the same direction as when they were natively presented on *S. typhimurium* and influenza, respectively, exposing relevant activation domains and B cell epitopes, respectively. Soluble protein expression of flagellin fused to SC was observed in *E. coli* (Figure S1A, Supporting Information). After purification, we achieved >90% purity for SC‐fused FliC (SC‐FliC) and FliCc (SC‐FliCc), and the predicted average molecular weight of ∼65.2 kDa (Figure S1B, Supporting Information; **Figure** **2**A). SC fused to H5 (SC‐H5) was expressed in a mammalian cell system, allowing for post‐translational glycosylation as in natively‐expressed H5,^[^ ^49^, ^50^ ^]^ which has been shown to be important in eliciting conformation‐dependent immune responses.^[^ ^51^, ^52^ ^]^

#### 2.1.2 Attachment of TLR5 Agonist (Flagellin) to NP Surface

The ST‐E2 protein NP allowed interior and exterior attachments designed for co‐delivery of adjuvants and antigens. To examine the display of, and activation by, flagellin on NPs, we attached each variant of flagellin onto the particles at two different surface densities. Conjugation of FliC and FliCc onto the surface of ST‐E2 yielded intact and monodisperse NPs (Figure 2; Figure S1, Supporting Information). As expected, when conjugated to either form of flagellin, the ST‐E2 monomer molecular weight increased from ∼30 kDa to ∼95 kDa (Figure 2A; Figure S1B,D, Supporting Information). Quantification revealed that 5.9 ± 1.1 and 21.2 ± 0.8 FliCc molecules per NP were conjugated for low‐density and high‐density NPs, respectively. The average hydrodynamic diameters of low‐density and high‐density loaded FliCc‐E2 NPs were 34 ± 1.2 nm and 46.2 ± 5.1 nm, respectively, with the size increase corresponding to the additional number of flagellin per NP (Figure 2B). Similar ratios and size changes were also obtained for FliC molecules attached to E2 NPs (Figure S1D,E, Supporting Information).

These NPs (low‐ and high‐density FliCc‐E2 and FliC‐E2 NPs) were used to evaluate the effects of loading TLR5 agonist on NPs for TLR5 activation in vitro. Stability assays of the SC‐fused flagellins (SC‐FliC and SC‐FliCc) showed that the cysteine‐stabilized flagellin fusion protein (SC‐FliCc) was more stable than the wild‐type fusion protein (SC‐FliC) (Figure S1C, Supporting Information); we therefore used SC‐FliCc for construction of NP vaccine formulations in subsequent in vivo studies.

#### 2.1.3 Attachment of TLR9 Agonist (CpG 1826) to NP Interior

The TLR9 agonist, CpG 1826, was conjugated to the interior of the ST‐E2 NP platform via an acid‐labile linker that allowed for CpG release in the endosome after uptake by antigen presenting cells (Figure 1).^[^ ^13^, ^39^ ^]^ The SDS‐PAGE band at ∼37 kDa supported the conjugation of one CpG molecule (∼7 kDa) to a ST‐E2 monomer, while the band at ∼30 kDa reflected unconjugated ST‐E2 monomer (Figure 2C). In addition, SDS‐PAGE of each CpG‐conjugated NP formulation (i.e., CpG‐ST‐E2, FliCc‐CpG‐E2, H5‐CpG‐E2, and H5‐FliCc‐CpG‐E2) showed no degradation and confirmed its chemical stability (Figure 2C). Sixty monomers self‐assembled into CpG‐ST‐E2 NPs, with no signs of aggregation and an average hydrodynamic diameter of 29.9 ± 2 nm (Figure 2D). Quantification indicated 16.1 ± 3.2 CpG 1826 molecules were encapsulated internally per 60‐mer ST‐E2 NP, which was consistent with our previously reported studies.^[^ ^13^, ^39^ ^]^

#### 2.1.4 Attachment of Antigen (Influenza H5 Hemagglutinin) to NP Surface

Conjugation of SC‐H5 antigen onto the surface of ST‐E2 yielded intact and monodispersed nanoparticles after optimization. When conjugating the NP with H5 antigen, we aimed for a minimum loading of six antigens per NP because antigen valences greater than ∼5 per NP are reported to be effective for optimal B cell receptor engagement and B cell activation.^[^ ^2^ ^]^ Upon SC‐H5 attachment, the ST‐E2 monomer molecular weight increased by ∼80–90 kDa, from ∼30 kDa to ∼110–120 kDa, consistent with the molecular weight of hemagglutinin conjugation (Figure 2C). Quantification estimated that 18.3 ± 1.2 H5 was conjugated to each E2 nanoparticle. H5‐E2 and H5‐CpG‐E2 hydrodynamic diameters were 48.2 ± 1.3 nm and 46.3 ± 1.1 nm, respectively (Figure 2D).

#### 2.1.5 Attachment of Flagellin and Hemagglutinin to Single NP

**Figure 3:**
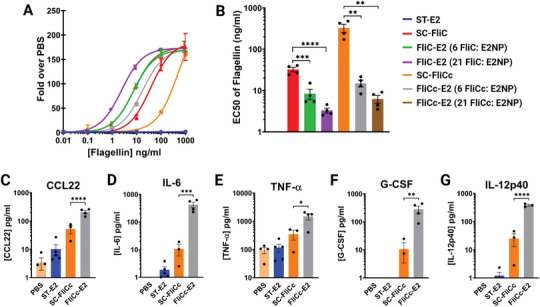
Conjugation of SC‐FliCc onto ST‐E2 NPs increases activation of TLR5 reporter cells and macrophages, relative to SC‐FliCc alone. A) Representative experiment of the activation of HEK‐blue hTLR5 cells after 16 h incubation. Absorbance of each group is normalized to the absorbance of PBS‐only incubation. EC50 is determined using a sigmoidal dose‐response curve‐fit of each group. B) Average EC50 concentrations of flagellin from HEK‐blue hTLR5 activation. Panels (C–G): Cytokine secretion from J774.1 macrophage cells after 24 h incubation. C) CCL22 (One PBS data point is between 0 and 1 pg mL^−1^). D) IL‐6 (PBS data are between 0 and 1 pg mL^−1^). E) TNF‐α. F) G‐CSF (PBS and ST‐E2 data are between 0 and 1 pg mL^−1^. One data point of SC‐FliCc is between 0 and 1 pg mL^−1^). G) IL‐12p40 (PBS data are between 0 and 1 pg mL^−1^. Three ST‐E2 data points are between 0 and 1 pg mL^−1^). Data in panel B is presented as an average ± SEM of four independent experiments (*n* = 4). Data in panels (C–G) is presented as an average ± SEM of at least three independent experiments (*n* ≥ 3). Statistical significance was determined by one‐way ANOVA followed by a Tukey multiple comparisons test. *p < 0.05, **p < 0.01, ***p < 0.001, and ****p < 0.0001.

Based on structural models predicting steric hindrance at the high surface densities and the flagellin activation results discussed below (**Figure** **3**), the low‐density flagellin‐E2 NP was used to ensure higher SC‐H5 conjugation numbers. We confirmed the simultaneous loading of nucleic acid (CpG) and two different proteins (SC‐FliCc and SC‐H5) on a single NP (Figure 2C, lane 10), along with several intermediate combinations of adjuvant and antigen attachment (Figure 2C). The complete dual‐adjuvant with antigen formulation (H5‐FliCc‐CpG‐E2) was quantified to have a H5:FliCc:CpG:E2 NP conjugation ratio of ≈17:6:16:1. Displaying both SC‐FliCc and SC‐H5 on the H5‐FliCc‐E2 and H5‐FliCc‐CpG‐E2 NPs further increased the diameters to 52.3 ± 0.3 nm and 50.3 ± 2.4 nm, respectively (Figure 2D). TEM images confirmed intact monodisperse NPs of the complete dual‐adjuvant with antigen particle (H5‐FliCc‐CpG‐E2) (Figure 2E). These stable NPs were then used in vivo to evaluate the prophylactic vaccine potential of simultaneous delivery of antigen and adjuvants uniformly attached to a single delivery vehicle.

### 2.2 Attachment of Flagellin onto Nanoparticles Increases Flagellin Bioactivity

Flagellin variants (SC‐FliC and SC‐FliCc) were conjugated to ST‐E2 NPs, and the resulting bioactivities were evaluated using a hTLR5 reporter cell line. SC‐FliC alone (not loaded on NPs) was approximately ten times more active than SC‐FliCc alone, which is consistent with previously reported results (**Figure** **3**A,B).^[^ ^32^ ^]^ Attaching SC‐FliC and SC‐FliCc on NPs at a low‐density of ∼6 flagellin (per ST‐E2 NP) significantly increased the activity of the flagellins, from average EC50s of ∼30 and ∼300 ng mL^−1^ (for unbound FliC and FliCc, respectively) to EC50s of ∼8 and ∼15 ng mL^−1^ (for FliC‐E2 and FliCc‐E2, respectively) (Figure 3A,B). At this low‐density surface loading, SC‐FliCc activity increased by more than an order of magnitude when NP‐bound and the disparity between SC‐FliC and SC‐FliCc was reduced from ∼10× to only ∼2×. This phenomenon of loading immunomodulatory motifs onto NPs has been shown to have significant benefits for cell recognition as increasing the valency and density of a motif can make receptor engagement more likely and activation stronger.^[^ ^2^, ^53^ ^]^

At high‐density loading of ∼21 flagellin per ST‐E2 NP, activation increased further to EC50s of ∼3 and ∼6 ng mL^−1^ for SC‐FliC and SC‐FliCc, respectively, but was not significantly higher than the response to the low‐density loaded NPs (Figure 3A,B). Like the low‐density NPs, by loading flagellin on NPs, the discrepancy in bioactivity between SC‐FliCc and SC‐FliC was significantly reduced to an extent that SC‐FliCc became nearly as active as SC‐FliC. Given this result, together with the higher observed stability of SC‐FliCc (Figure S1C, Supporting Information), subsequent studies utilized SC‐FliCc at the lower surface density.

### 2.3 FliCc‐E2 Nanoparticles Promote Inflammatory Cytokine Release By Macrophages

To examine the effect of loading flagellin on NPs in a more immunologically‐relevant cell type that expresses TLR5, we examined macrophage activation by incubating cells with flagellin (unbound SC‐FliCc and low‐density FliCc‐E2 NPs) and quantifying secreted inflammatory cytokines. We consistently observed activation of five cytokines (CCL22, IL‐6, TNF‐α, G‐CSF, and IL‐12p40), which overlapped with the cytokine production reported from previous studies utilizing this macrophage cell line for flagellin activation (IL‐6, TNF‐α, GCSF, and IL‐1β).^[^ ^54^, ^55^, ^56^ ^]^ In our investigation, inflammatory cytokine secretion was significantly increased by conjugation of SC‐FliCc to ST‐E2 NPs, relative to SC‐FliCc alone (Figure 3).

Interestingly, these observed cytokines are associated with both innate and adaptive immunity. Specifically, CCL22 production increased by ∼4×, from an average of 53 pg mL^−1^ (for free SC‐FliCc) to 210 pg mL^−1^ when SC‐FliCc was attached to the NP (FliCc‐E2; Figure 3C). CCL22 is attributed to adaptive immune responses as a chemoattractant for T cells and dendritic cells, and it is a regulator of Th2‐type immune responses.^[^ ^57^, ^58^ ^]^ After conjugating SC‐FliCc onto the NP, IL‐6 production increased by 40×, from 10.6 pg mL^−1^ (free SC‐FliCc) to 425 pg mL^−1^ (FliCc‐E2). IL‐6 is connected to both proinflammatory and anti‐inflammatory immune responses, and it is often recognized as an acute phase response regulator and stimulator of adaptive immune responses.^[^ ^59^ ^]^ TNF‐α, a macrophage regulator and acute inflammation activator,^[^ ^60^, ^61^ ^]^ also increased from 350 to 1490 pg mL^−1^. G‐CSF, an innate immune system cytokine that regulates neutrophil maturation,^[^ ^62^, ^63^ ^]^ increased by nearly 30×, from 10.7 to 280 pg mL^−1^ for cells incubated with SC‐FliCc and FliCc‐E2, respectively. IL‐12p40 production increased by more than 10×, from 25 pg mL^−1^ secreted by SC‐FliCc alone to 380 pg mL^−1^ secreted when SC‐FliCc was loaded on the NP. IL‐12p40 is a cytokine that is part of both the innate and adaptive immune response as a macrophage chemoattractant and promoter of T cell differentiation and proliferation.^[^ ^64^, ^65^, ^66^ ^]^ Notably, IL‐1β levels were below detection in our activation studies. In all this, unique cytokine profile is consistent with the many observations of flagellin eliciting Th1 and/or Th2 responses.^[^ ^30^, ^67^, ^68^ ^]^

### 2.4 Attaching H5 Hemagglutinin onto Nanoparticles Elicits Significantly Higher IgG Responses than Soluble SC‐H5 Alone

**Figure 4:**
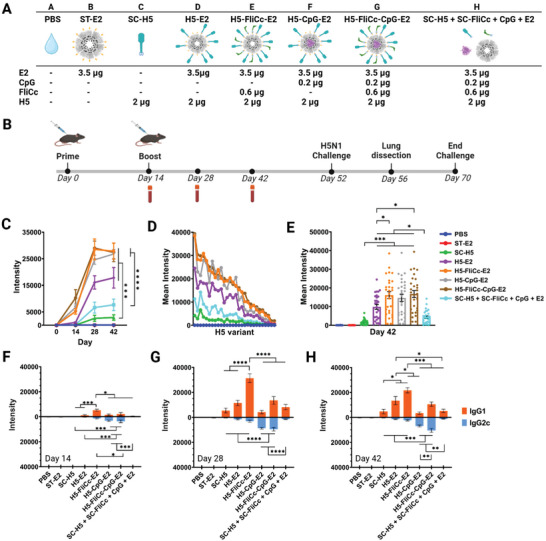
Antibody response elicited by H5‐FliCc‐CpG‐E2 dual‐adjuvant nanoparticle formulations. A) Table describing each formulation and its individual components. The molar amounts of each component are shown in Figure S2, Supporting Information. SC‐H5 is colored blue, SC‐FliCc is green, CpG is magenta, and ST‐E2 NP is grey. To avoid conjugation from occurring during immunization, the E2 NP used in group H did not have SpyTag. B) Immunization schedule and challenge timeline. C) IgG response to H5 variant A/Vietnam/1194/2004 in plasma on days 0, 14, 28, and 42. D) Homosubtypic IgG response to 28 different variants of H5, from day 42 plasma. Each column of spots corresponds to the antibody response to a unique H5 antigen variant, with each spot being an average response from *n* = 8 mice. E) Box plot of homosubtypic IgG response to H5 variants at day 42. Each spot corresponds to response to a different H5 variant (*n* = 8 mice). Plotted is average ± SEM of 28 variants. F) IgG1/IgG2c response to H5 variant A/Vietnam/1194/2004 on day 14. G) IgG1/IgG2c response to H5 variant A/Vietnam/1194/2004 on day 28. H) IgG1/IgG2c response to H5 variant A/Vietnam/1194/2004 on day 42. Data in panel (C) is presented as an average ± SEM of eight individual mice (*n* = 8). Statistical significance was determined by one‐way ANOVA, followed by a Tukey multiple comparisons test of day 42 data. Data in panel (E) is presented as an average ± SEM of individual variants (*n* = 28). Statistical significance was determined by one‐way ANOVA, followed by a Tukey multiple comparisons test. Data in panels (F–H) is presented as an average ± SEM of eight individual mice (*n* = 8). Statistical significance was determined by one‐way ANOVA, followed by a Tukey multiple comparisons test.*p < 0.05, **p < 0.01, ***p < 0.001, and ****p < 0.0001.

We investigated the antibody response elicited by different vaccine formulations after a prime and boost immunization in C57Bl/6 mice (**Figure** **4**A,B). The study included different combinations of adjuvant (flagellin, CpG), HA antigen (H5), and bound (or unbound) to the E2 NP, described in Figure 4A. Sera from each animal was probed against HA protein microarrays displaying 28 variants of H5 (Figure S3 and Table S1, Supporting Information) including the subtype H5 used in the vaccine formulations and immunizations (A/Vietnam/1194/2004). Immunizing with the SC‐H5 antigen alone (no NP) elicited the lowest H5‐specific IgG response after 42 days (Figure 4C). Strikingly, when SC‐H5 was displayed on ST‐E2 nanoparticles (H5‐E2 NPs), with or without the TLR adjuvants, H5‐specific IgG antibody response significantly increased relative to immunization with SC‐H5 alone (Figure 4C). H5‐E2 NPs elicit over five times greater IgG antibody responses toward H5 by simply displaying the antigen on the NP scaffold. H5‐E2 also elicited observably higher H5‐specific IgG responses compared to the formulation with all antigen and adjuvant vaccine components present but unconjugated (SC‐H5 + SC‐FliCc + CpG + E2 [without ST]) (Figure 4C). This demonstrates that simply displaying antigen on a NP can produce adjuvating magnitudes similar to, or even higher than, having two highly specific PRR targets (flagellin and CpG) soluble in the formulation. It has been previously reported that NP size and repetitive antigen display can play a crucial role in B cell receptor engagement and B cell activation.^[^ ^2^, ^69^, ^70^ ^]^ Our data supports this premise that B cell activation can be augmented by the decoration of NPs with repetitive epitopes which mimic natural pathogens such as viruses and can yield strong innate responses by increasing uptake from antigen‐presenting cells and enabling binding and simultaneous activation of multiple B cell receptors.^[^ ^2^, ^71^ ^]^

The effect of any antibodies produced toward the NP conjugation system itself is often a concern. Others using virus‐like particle (VLP) platforms via ST/SC have seen antibodies produced to SpyCatcher and NP scaffolds. However, after surface conjugation of antigens, there was reduced immunogenicity to ST/SC and NP platform (relative to SC or scaffold with no antigens), with successful induction of prophylactic immune responses even after multiple immunizations.^[^ ^72^, ^73^ ^]^ It appears that the antigen on the NP surface reduces access to SC and the VLP. In our studies, even if E2 antibodies are generated, they are not observed to neutralize the antigen‐specific immune responses elicited, as demonstrated by the elevated antibody responses shown here and efficacy after lethal challenge with H5N1 influenza virus shown later in this investigation.^[^ ^12^, ^13^, ^40^ ^]^

### 2.5 One or Two Adjuvants Attached to a Nanoparticle Elicit Significantly Higher IgG Responses Than Two Co‐Administered Soluble Adjuvants

We examined the effects of attaching the adjuvants to the NP vaccines, compared to unconjugated adjuvants. Typically, 1–100 µg CpG and 0.5–10 µg flagellin have been used as soluble adjuvants for in vivo immunizations.^[^ ^11^, ^31^, ^37^, ^40^, ^74^, ^75^, ^76^, ^77^ ^]^ In each of our formulations, the dosages of CpG and flagellin were ∼0.2 and ∼0.6 µg, respectively, both of which lay at the low end of dosage amounts for typical immunization studies. The total IgG responses obtained with CpG‐E2‐ and FliCc‐E2‐based formulations (H5‐CpG‐E2 and H5‐FliCc‐E2, respectively) were relatively high and comparable to one another (Figure 4C). Unexpectedly, the combination of CpG and flagellin both loaded together onto one NP (H5‐FliCc‐CpG‐E2) did not increase the total IgG response above the effects of a single adjuvant loaded onto the NP (H5‐CpG‐E2 or H5‐FliCc‐E2), despite being conjugated to agonists for two different TLR receptors. This may be in part due to both adjuvants primarily signaling through the MyD88 pathway to activate NFκB leading to the secretion of inflammatory cytokines.^[^ ^78^ ^]^

Interestingly, loading a single adjuvant onto a NP (H5‐CpG‐E2 or H5‐FliCc‐E2) or both adjuvants onto a NP (H5‐FliCc‐CpG‐E2) elicited significantly stronger IgG responses than having the equivalent amounts of both soluble adjuvants dosed concurrently (SC‐H5 + SC‐FliCc + CpG + E2) (Figure 4C). Encapsulating CpG in a particle had been shown to activate antigen presenting cells at significantly lower concentrations than unbound CpG, indicating the advantage for CpG‐NP conjugation for eliciting increased immune response.^[^ ^39^ ^]^ Other studies have also shown that NPs capable of simultaneously delivering flagellin or CpG, together with antigen, can increase the immune response mounted against the target antigen.^[^ ^13^, ^29^, ^40^ ^]^ In our formulations, CpG was encapsulated within a NP to increase uptake efficiency of CpG, and flagellin was displayed onto a NP to increase TLR5 receptor engagement of flagellin, both properties of which could increase the dose of CpG and flagellin that an individual cell received upon interacting with the E2 NP (relative to unbound CpG or flagellin).

### 2.6 E2‐Bound Formulations Elicited Higher Homosubtypic Cross‐Reactivity Amongst H5 Variants (Relative to Unbound Antigen or Adjuvants)

The breadth of the antibody response elicited by the vaccine formulations was examined by quantifying subtype cross‐reactivity on the protein microarrays. We demonstrated that attaching SC‐H5 on ST‐E2 NPs (i.e., H5‐E2) enhanced antibody breadth relative to unbound SC‐H5. Shown in Figure 4D,E are IgG profiles for day 42 sera toward 28 variants of H5 (variants listed in Table S1, Supporting Information). The plot in Figure 4D shows response intensities for each vaccine group (mean of *n* = 8 mice) against all H5 hemagglutinins printed on the microarray, spanning H5 variants 1 through 28 (left to right on horizontal axis). The data for individual H5 variants are also shown in the box plots in Figure 4E. Displaying SC‐H5 on ST‐E2 NPs (H5‐E2) not only significantly increased homosubtypic cross‐reactivity relative to SC‐H5 alone (*p* < 0.001) (Figure 4D,E), but also elicited significantly higher cross‐reactivity than SC‐H5 co‐administered with unbound CpG and SC‐FliCc (SC‐H5 + SC‐FliCc + CpG + E2) (p < 0.05) (Figure 4D,E). All NP formulations with single or both adjuvants conjugated also elicited significantly higher homosubtypic cross‐reactivities than the unconjugated dual adjuvant formulation (SC‐H5 + SC‐FliCc + CpG + E2) (Figure 4D,E). This demonstrates that although the same amount of adjuvant was administered in vivo in these studies, attachment to the E2 NP significantly increased their effect on the immune response at this dose.

The addition of CpG onto H5‐E2 NPs (H5‐CpG‐E2) increased the average of its homosubtypic cross‐reactivity compared to H5‐E2 NPs alone; however, only when SC‐FliCc was attached (H5‐FliCc‐E2 and H5‐FliCc‐CpG‐E2), did its homosubtypic cross‐reactivity significantly increase above H5‐E2 NPs alone (*p* < 0.05) (Figure 4E). Homosubtypic cross‐reactivity of antibodies generated by the H5 (A/Vietnam/1194/2004) vaccine is mediated by B cell clones that recognize shared epitopes across drift variants.^[^ ^79^ ^]^ Homosubtypic cross‐reactivity produced by the H5−E2 vaccine reported here was significant as it may offer a path to providing protection against drift variants. Current seasonal influenza vaccines elicited antibodies that were highly specific to the immunizing variant;^[^ ^80^, ^81^, ^82^ ^]^ consequently, seasonal vaccines need to be modified each year in response to antigenic drift. Avian influenza H5N1 is endemic in wild birds. It is also known to cause sporadic zoonotic infections in humans, and therefore has potential to cause pandemics.^[^ ^43^, ^44^ ^]^ A vaccine able to provide broader protection than conventional inactivated or attenuated virus formulations, accomplished through the use of adjuvants or nanoparticles, would reduce the need for annual reformulations in the case of seasonal vaccines and improve anticipatory protection against potentially emerging pandemic influenzas.

### 2.7 IgG1 and IgG2c Antibody Responses Can Be Modulated by Adjuvant Type and Attachment on Nanoparticle

Sera on days 14, 28, and 42 were probed using microarrays for H5‐specific IgG1 and IgG2c using isotype‐specific secondary antibodies (Figure 4F–H). Antibody class switching to IgG1 or IgG2c is frequently used as a surrogate marker for Th2 and Th1 immune responses, respectively.^[^ ^13^, ^83^, ^84^, ^85^ ^]^ Profiles of the IgG1 and IgG2c antibody responses suggest modulation capabilities that depend on the adjuvant used and whether it is loaded on the E2 nanoparticle (Figure 4F–H). Signs of IgG1/IgG2c response skewing were observed as early as day 14 for E2 NP formulations after only a prime immunization, while SC‐H5 antigen alone or soluble co‐administration of SC‐H5, SC‐FliCc, CpG, and E2 did not elicit strong enough responses for detection (Figure 4F). By day 28, the IgG1/IgG2c responses of each formulation were heightened, with day 42 responses being comparable in magnitude while maintaining subclass skewing (Figure 4G,H). The only sign of change from day 28 to day 42 was a decrease in the IgG1 response of the H5‐FliCc‐E2 NP immunized group. Interestingly, although its IgG1 response decreased from day 28 to day 42, its total IgG response (Figure 4C) did not change. One possible explanation for this IgG1 response decrease, while maintaining total IgG, was that IgG may be switching to another subclass, such as IgG3 or IgG2b.

To characterize the IgG1/IgG2c bias for each formulation, we focused on data from day 42. Soluble SC‐H5 antigen elicited a weak total IgG response that was biased toward IgG1 (Th2) (Figure 4H). Loading SC‐H5 onto NPs (H5‐E2) significantly increased total IgG and retained the IgG1‐bias that was observed for SC‐H5 alone (Figure 4H). The addition of SC‐FliCc conjugated to the H5‐E2 NP (H5‐FliCc‐E2) further significantly increased IgG1 production without altering Th2 bias (Figure 4H). Flagellin has been utilized as an adjuvant in numerous vaccine formulations and has been shown to elicit Th1‐ and/or Th2‐type responses.^[^ ^35^, ^36^, ^37^, ^86^, ^87^ ^]^ The exact mechanisms for the reasons that flagellin elicited a Th1 or Th2 bias response are still being studied, but some have attributed these observations to be antigen‐specific or dependent on certain cell‐specific stimulations by flagellin.^[^ ^30^, ^54^, ^86^, ^87^, ^88^, ^89^, ^90^ ^]^ In our hands, it appeared that flagellin did not change the baseline Th2 bias elicited by SC‐H5 alone and the H5‐E2 NP. In contrast, when CpG was internally loaded into the ST‐E2 NP (H5‐CpG‐E2), the total IgG response significantly increased (relative to SC‐H5 antigen alone) and the response shifted toward Th1 bias, with significant decrease of IgG1 and significant increase of IgG2c, compared to H5‐E2 NPs alone (Figure 4H). The Th1‐skewing property of CpG was consistent with other studies.^[^ ^91^, ^92^, ^93^, ^94^ ^]^ In our previous studies, we also showed the capacity of E2 NPs to elicit CD8+ mediated anti‐tumor immunity when conjugated with tumor peptide antigens and administered with CpG.^[^ ^11^, ^12^ ^]^

Given the Th2 and Th1 profiles observed from using SC‐FliCc and CpG, respectively, it was hypothesized that combining the two adjuvants together may give a more balanced Th2/Th1 response. Surprisingly, when soluble SC‐H5 antigen and both adjuvants were co‐administered without conjugation (SC‐H5 + SC‐FliCc + CpG + E2), the antibody response magnitude and immune response biases did not differ significantly from immunizing with SC‐H5 antigen alone (Figure 4H). This could be that the adjuvant dosages administered here were too low to yield a response for the unconjugated soluble forms.^[^ ^11^, ^31^, ^37^, ^40^, ^74^, ^75^, ^76^, ^77^ ^]^ However, when all components of the vaccine formulation were conjugated to the ST‐E2 NP (H5‐FliCc‐CpG‐E2), a distinctive immune response was observed. Having both flagellin and CpG conjugated to the nanoparticle elicited a balanced IgG1/IgG2c response (Figure 4H). Although antibody responses have conventionally been the focus of evaluating influenza vaccine efficacy, more recent studies have shown that cell‐mediated responses are also valuable.^[^ ^95^, ^96^, ^97^ ^]^ T cells can recognize epitopes that can be highly conserved between variants located within the structure of antigens. Our data shows the ability of our NP platform to skew IgG responses toward IgG2c, suggesting a stronger Th1 CD4+ T cell‐mediated response, which may have benefits in the context of influenza vaccine design.^[^ ^96^, ^98^ ^]^ The ability to precisely modulate the ratio of Th1/Th2 immune response by using a NP platform conjugated with different TLR agonists is novel and significant, as the majority of adjuvants used in FDA‐approved human vaccines primarily stimulate one type of immunity;for example, the most popular adjuvant is alum, which primarily stimulates Th2‐biased immunity.^[^ ^78^, ^99^ ^]^

### 2.8 E2 Nanoparticle Formulations Protect Mice from Lethal H5N1 Influenza Challenge

**Figure 5:**
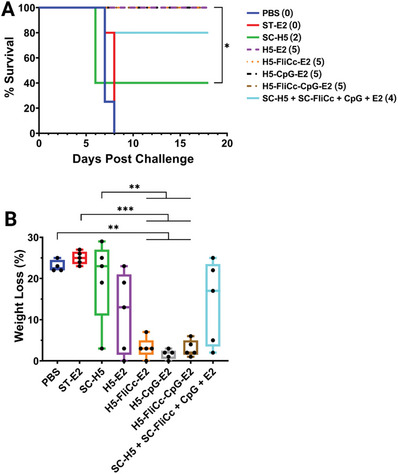
Immunization with nanoparticles bound to H5 and TLR adjuvants protect mice from the lethal challenge of influenza and improve morbidity. A) Survival curves after challenge with H5N1 influenza on day 52 of immunization. The numbers in parentheses in the legend (i.e., (0), (2), and (5)) show the number of mice that survived for each vaccination group. All groups with components conjugated to nanoparticles yielded 100% survival (i.e., H5‐E2, H5‐FliCc‐E2, H5‐CpG‐E2, and H5‐FliCc‐CpG‐E2). B) Morbidity plot showing maximal weight loss of each mouse after viral challenge. Data in each group reflects *n* ≥ 4 individual mice. Statistical significance was determined by one‐way ANOVA, followed by a Tukey multiple comparisons test. Mantel–Cox log‐rank test used for survival curve analysis. *p < 0.05, **p < 0.01, and ***p < 0.001.

Thirty‐eight days after the final immunization, mice (*n* = 8 per group) were inoculated with a lethal dose of H5N1 virus expressing the A/Vietnam/1194/2004 H5 variant (Figure 4B). The mice were subsequently monitored for changes in weight, physical appearance, and behavior. Animals that lost greater than 20% of their original body weight were euthanized, and weight data of each individual mouse is shown in Figure S4, Supporting Information. Four days after the start of the challenge, the lungs of three mice per group were harvested for viral lung titers. Mice administered PBS and the ST‐E2 NP alone (no H5 antigen, no TLR agonists) succumbed to infection (**Figure** **5**A). Mice immunized with the SC‐H5 antigen alone showed 40% survival, and with the addition of soluble flagellin and CpG (SC‐H5 + SC‐FliCc + CpG + E2) showed 80% survival. All E2 NPs bound to antigen, including the unadjuvanted H5‐E2 NP, demonstrated 100% survival from lethal H5 influenza challenge (Figure 5A). Despite having immune responses that skewed differently (Th1 or Th2), each E2 formulation was capable of protecting the mice. We postulate that although the T cell response (Th1 vs Th2) from a vaccination may vary between different adjuvants, efficacy was accomplished predominantly by neutralizing antibodies.

Morbidity, manifested as transient weigh loss (or “partial protection”) before regaining weight, helped reveal the nuances of the E2 formulations. Mice immunized with E2 NPs conjugated with adjuvant (i.e., H5‐FliCc‐E2, H5‐CpG‐E2, and H5‐FliCc‐CpG‐E2) exhibited little to no signs of morbidity as seen by the minimal weight loss (Figure 5B). Viral lung titers showed a similar trend for NPs conjugated with adjuvant (Figure S5, Supporting Information). Nanoparticles without adjuvant (H5‐E2), interestingly, performed similarly to that of the unconjugated complete formulation (SC‐H5 + SC‐FliCc + CpG + E2) with some mice exhibiting noticeable weight loss (Figure 5B). These observations demonstrated that to have 100% survival and minimal morbidity, formulations must have conjugated adjuvant (either singular or dual) on nanoparticles. The additive value to protection observed from the modular additions to the NP construction (i.e., from SC‐H5 to H5‐E2 to H5‐FliCc‐E2) demonstrates the control we have in modulating the immune response.

## 3 Conclusion

The co‐delivery of a model protein antigen (H5 hemagglutinin) and two adjuvants (flagellin and CpG) on a single NP was successfully synthesized using the E2 protein nanoparticle scaffold and the SpyTag/SpyCatcher bioconjugation system. This strategy yielded stable monodispersed NPs with H5 and flagellin displayed on its exterior and CpG loaded in its internal cavity. Displaying as little as six flagellin molecules on the ST‐E2 NP significantly increased the bioactivity of flagellin and increased the activation of immunologically relevant cells in vitro by upward of an order of magnitude compared to unconjugated flagellin. SC‐H5 alone was weakly immunogenic and elicited a Th2 bias response. Conjugation of SC‐H5 onto ST‐E2 NPs (H5‐E2) significantly enhanced magnitude and breath of antibody response but did not change the underlying Th2 profile. Compared to unconjugated soluble adjuvants co‐administered with antigen (SC‐H5 + SC‐FliCc + CpG + E2), conjugation of adjuvants onto H5‐E2 NPs increased both antibody magnitude and breadth, showing that adjuvant conjugation to NP was necessary to maximize the adjuvant activity.

IgG1/IgG2c antibody subclassing could also be precisely modulated, dependent on the adjuvant and whether it was attached to the nanoparticle. Addition of TLR agonist flagellin elevated magnitude and breadth but did not affect Th2 profile, while addition of CpG also enhanced magnitude and breadth but polarized the response into a Th1 direction. Interestingly, when both flagellin and CpG were loaded on the NP vaccine, a more balanced IgG1/IgG2c response was observed, suggesting the generation of both Th1 (associated with cellular immunity) and Th2 (associated with humoral immunity) responses. Mice immunized with any E2 NP‐based vaccine exhibited complete protection from H5N1 influenza challenge. Notably, only mice that received adjuvanted E2 NP vaccine showed minimal or no signs of sickness. Our successful engineering of a protein nanoparticle to precisely orient and attach antigen and multiple adjuvants enabled specific modulation of an immune response. This highlights the potential of nanoparticle‐based delivery systems for the development of prophylactic vaccines, which could offer broader protection, reduce the need for annual reformulations of seasonal vaccines, and improve anticipatory protection against emerging pandemic pathogens.

## 4 Experimental Section

### Materials

Chemical and cloning reagents were purchased from Fisher Scientific or New England Biolabs (NEB) unless otherwise noted. DH5α and BL21(DE3) *E. coli* were used for general cloning and expression studies, respectively. DNA minipreps and extractions were performed with the QIAprep Spin Miniprep Kit (Qiagen) and GeneJET Gel Extraction Kit (Thermo Fisher Scientific), respectively. DNA primers were synthesized and ordered from Integrated DNA Technologies (IDT). CloneJET PCR cloning kit (Thermo Fisher Scientific) was used for all polymerase chain reactions (PCRs). Plasmid pET11a was used as the expression vector for all protein constructs. HA variants used in the protein arrays were purchased from Sino Biological.^[^ ^100^ ^]^

### Construction of SpyCatcher‐Flagellins and SpyCatcher‐Hemagglutinin Fusion Proteins

The plasmids encoding the wild type flagellin (FliC) and the cysteine‐stabilized flagellin (FliCc) were previously synthesized and generously gifted by Dr. James Swartz.^[^ ^32^ ^]^ The plasmid containing the SpyCatcher gene (pDEST14‐SpyCatcher) was obtained from Addgene. The plasmid containing the hemagglutinin subtype H5 gene was synthesized using RNA obtained from H5N1 virus A/Vietnam/1194/2004 (National Institute for Biological Standards & Controls, South Mimms, UK; catalog # NIBRG‐14), which was used as a template for cDNA synthesis. In brief, 140 µL allantoic fluid from H5N1 infected hen eggs was processed using the QIAamp Viral RNA minikit (Qiagen). To synthesize cDNA of the H5 A/Vietnam/1194/2004 gene from RNA, the forward primer: 5′‐TTTGCAATAGTCAGTCTTGTTAAAAGTG‐3′ and reverse primer: 5′‐AATTCTGCATTGTAACGACCC‐3′ were used. cDNA of the H5 A/Vietnam/1194/2004 gene was then inserted into a pJET cloning vector using a CloneJET PCR cloning kit. To fabricate the well‐studied N‐terminus truncated form of SpyCatcher (referred to here as SC), the first 24 AA of the native SpyCatcher was deleted.^[^ ^101^, ^102^ ^]^ To accomplish this, forward primer #1 (introduce deletion and TEV cleavage site) 5′‐GATTACGACATCCCAACGACCGAAAACCTGTATTTTCAGGGCGATAGTGCTACCCATATTAAATTCTCAAAACG‐3′, forward primer #2 (introduce 6x‐HisTag and endonuclease cut‐site) 5′‐CATATGTCGTACTACCATCACCATCACCATCACGATTACGACATCCCAACGACCG‐3′, and reverse primer 5′‐GCTAGCAATATGAGCGTCACCTTTAGTTGCTTTGCC‐3′ were used. To introduce the endonuclease sites and GS‐rich spacer on both FliC and FliCc variants for fusion to SpyCatcher, the forward primer was 5′‐ ATATGCTAGCATGGGATCAGGGGGATCAGGTGGCAGCGGAGCACAAGTGATTAATACAAACAGCCTGTCGC‐3′ and 5′‐ATATGCTAGCATGGGATCAGGGGGATCAGGTGGCAGCGGAATACAAGTGATTAATACAAACAGCCTGTCGC‐3′, respectively, and the reverse primer was 5′‐ATATGGATCCTTAACGCAGTAAAGAGAGGACGTTTTGC‐3′. To introduce the endonuclease sites and GS‐rich spacer on H5 hemagglutinin for fusion to SpyCatcher, the forward primer was 5′‐GCTAGCGGTTCAGGAACAGCAGGTGGTGGGTCAGGTTCCGATCAGATTTGCATTGGTTACCATG‐3′ and the reverse primer was 5′‐GGATCCTTATATTTGGTAAATTCCTATTGATTCCAATTTTAC‐3′. The SC‐H5 fusion protein gene was cloned into a pJET vector. A standard phusion high‐fidelity DNA polymerase protocol was used for PCRs, and sequences were confirmed by Azenta (Table S2, Supporting Information). The protein was expressed by BioTimes Inc. in a mammalian CHO cell system.

### Expression, Purification, and Characterization of SpyCatcher‐Flagellins and SpyCatcher‐Hemagglutinin

The SpyCatcher‐flagellins were prepared similarly to previously described SpyCatcher fusion proteins.^[^ ^13^, ^103^ ^]^ Proteins were expressed in *E. coli* as follows: after growing cells to an OD of 0.6–0.9 and induction with 1 mm IPTG for 3 h at 37 °C, the cells were pelleted and stored at −80 °C before lysing. Cells were lysed via French press, and soluble protein was purified using a HisPur Ni‐NTA resin batch protocol (ThermoFisher Scientific). In brief, soluble cell lysates were mixed with equal parts equilibration buffer and applied to a HisPur Ni‐NTA affinity spin column using a packing ratio of 1.5 mL of resin per 10 mL of lysate slurry. The lysate incubated with the resin for 1 h at 4 °C. Wash buffers and elution buffer containing 75 and 150 mM imidazole, and 250 mM imidazole, respectively, were used to attain pure SC‐flagellins. Pure protein fractions were collected, and buffer was exchanged into PBS to remove imidazole using an Amicon Stirred Cell unit with a 10 kDa MWCO Ultracel membrane. The purified protein was characterized by mass spectrometry (Xevo G2‐XS QTof) and SDS‐PAGE, and bicinchoninic acid assay (BCA) for molecular weight and purity, and protein concentration, respectively. LPS was removed following the protocol used for the E2 protein.^[^ ^13^ ^]^ Briefly, Triton X‐114 was added to the purified protein at 1% v/v, chilled to 4 °C, vortexed, and heated to 37 °C. The mixture was then centrifuged at 18 000 × *g* and 37 °C for 1 min, and the protein containing aqueous phase was separated from the detergent phase. This total process was repeated nine times. Residual Triton was removed with detergent removal spin columns. LPS levels were below 0.1 EU per µg of SC‐flagellin protein and were determined by an LAL ToxinSensor gel clot assay (Genscript).

Expression and purification of SpyCatcher‐H5 was performed by BioTimes Inc. in a mammalian Chinese hamster ovary (CHO) cell system. The resulting fusion protein was purified to >90% using Ni‐affinity chromatography and was concentrated to 1 mg mL^−1^ in PBS, with an endotoxin level < 0.1 EU per µg of protein, as determined by the LAL assay (Genscript).

### CpG and SpyCatcher Conjugation onto SpyTag‐E2 Particles

The TLR9 agonist CpG 1826 (5′‐tccatgacgttcctgacgtt‐3′) (CpG) was synthesized with a phosphorothioated backbone and 5′ benzaldehyde modification by Integrated DNA Technologies (IDT). CpG was conjugated to the internal cavity of the ST‐E2 nanoparticle using a hydrazone‐forming linker as described previously.^[^ ^39^ ^]^ In brief, the internal cavity cysteines of ST‐E2 were reduced with TCEP for 30 min, followed by incubation with the N‐(β‐maleimidopropionic acid) hydrazide (BMPH) linker for 2 h at room temperature (RT). Unreacted linker was removed using 40 kDa cutoff Zeba spin desalting columns (Pierce). The aldehyde‐modified CpG was subsequently added and incubated overnight at RT to allow for internal cavity conjugation after diffusion through the nanoparticle's pores. Unreacted CpG was removed by desalting spin columns. Conjugation was estimated by SDS‐PAGE and measured by band intensity analysis.

Directly incubating SpyCatcher‐flagellins and SpyCatcher‐H5 with SpyTag‐E2 particles allowed for spontaneous isopeptide bond formation and conjugation. SpyCatcher‐flagellins were incubated with ST‐E2 NPs at a 0.1:1 and 0.4:1 (SC‐flagellin:ST‐E2 monomer) molar ratio for 22 h at 4 °C to synthesize low‐ and high‐density NPs, respectively. To synthesize H5‐loaded NPs, SpyCatcher‐H5 was incubated with ST‐E2 NPs at a 0.3:1 (SC‐H5:ST‐E2 monomer) molar ratio, supplemented with 500 mm NaCl, for 22 h at 4 °C. SDS‐PAGE densitometry analysis with protein standards was used to quantify protein loading onto the particles. Dynamic light scattering (DLS; Malvern Zetasizer Nano‐ZS) and transmission electron microscopy (TEM) were used to measure the size, assembly, and monodispersity of the particles. Transmission electron micrographs, on Cu 200 mesh carbon coated grids (Electron Microscopy Sciences) with 2% uranyl acetate‐stained nanoparticles, were obtained on a JEM‐2100F (JEOL) instrument with a Gatan OneView camera (Gatan).

### In Vitro Characterization of Flagellin Bioactivity

To characterize flagellin bioactivity, the HEK‐blue hTLR5 reporter cell line (Invivogen), which overexpresses human TLR5 on its surface and contains an inducible secreted alkaline phosphatase (SEAP) gene, was used. The manufacturer's protocol was followed using HEK‐blue detection media to evaluate activation. Briefly, in a 96‐well tissue culture plate at ∼25 000 cells per well, concentrations of flagellin ranging between 0.01 and 1000 ng mL^−1^ were added and incubated with HEK‐blue detection media at 37 °C in a CO_2_ incubator for 16 h. The enzymatic activity of SEAP was measured using a spectrophotometer plate reader (SpectraMax M2) by absorbance at 630 nm.

To access the activity of flagellin in a more immunologically‐relevant cell, the macrophage cell line J774.1 (UCSF cell bank) was employed. Cells were plated at ∼100 000 cell per well in 96 well plates and stimulated with 5 ng mL^−1^ flagellin for 24 h. The supernatant of cell culture was collected, and the concentration of cytokines (CXCL1 [KC], TGF‐β1 [Free Active Form], IL‐18, IL‐23, CCL22 [MDC], IL‐10, IL‐12p70, IL‐6, TNF‐α, G‐CSF, CCL17 [(TARC], IL‐12p40, IL‐1β) was measured by a LEGENDplex Mouse Macrophage/Microglia Panel (13‐plex) (BioLegend), following the manufacturer's instructions.

### Mice, Immunizations, and Challenge

All animal work was approved by the UCI Institutional Animal Care and Use Committee (IACUC #AUP‐18‐096) and by the Animal Care and Use Review Office (ACURO) of the U.S. Army Medical Research and Materiel Command (USAMRMC). The laboratory animal resources at UCI were internationally accredited by the Association for Assessment and Accreditation of Laboratory Animal Care (AAALAC #000238). All virus handling was performed in USDA inspected and approved BSL2+/ABSL2+ facilities. Female C57Bl/6 mice were purchased from Charles River Inc. and housed in standard cages with enrichment. Briefly, 6–8 week old female C57BL/6 mice (eight mice per group) were immunized with 50 µL vaccine formulations via the subcutaneous route (left flank) according to the dosages and schedule shown in Figures 4A,B, respectively. A total dosage of 4 µg of hemagglutinin antigen (2 µg each for prime and boost) was chosen for this study, which fell with the typical range of 2–15 µg used for influenza‐based vaccination studies in mice.^[^ ^42^, ^100^, ^104^, ^105^, ^106^ ^]^ The mice were weighed daily for 2 weeks after each injection and monitored for any changes in behavior or appearance. On days 14, 28, and 42, blood was collected via cheek vein into heparinized tubes and plasma stored at −80 °C until required for use. On day 52 of the study, transiently anesthetized mice were administered 10^4^ TCID50 mL^−1^ in a volume of 50 µL of virus (A/Vietnam/1194/2004 reassortant virus preparation NIBSC, NIBRG‐14) via the intranasal route. Mice were monitored daily for behavior and body weight until the endpoint, defined when > 20% of the original body weight was lost or 18 days, whichever occurred sooner. In addition, on day 4 post challenge, the lungs of three mice were harvested for viral lung titers by qPCR. Morbidity (transient weight loss or “partial protection”) was defined as maximum weight drop after viral challenge.

Lungs of infected mice (three per group) were harvested 4 days post infection, and lung viral titers were subsequently quantified using qPCR. Briefly, for total RNA extraction, lungs were weighed, mixed with 1 mL of Trizol (Thermo Fisher Scientific), and homogenized using a GentleMacs Tissue Homogenizer (Miltenyi Biotec) applying the RNA‐01 program. Then, the total RNA was extracted using Phasemaker‐TM tubes following the manufacturer's recommendation (Thermo Fisher Scientific). The RNA sample was stored at −70 °C until its use for RT‐qPCR. HA5 gene quantification by qPCR was performed based on the World Health Organization information for the molecular detection of influenza viruses, protocol 3 with slight modifications (February 2021). For HA5 gene amplification primers H5HA‐205‐227v2‐For 5′‐CGATCTAGATGGAGTGAAGCCTC‐3′, H5HA‐326‐302v2‐Rev 5′‐CCTTCTCCACTATGTAAGACCATTC‐3′, and the TaqMan probe H5‐Probe‐239‐RVa2 5′‐56‐FAM‐TGTAGTTGA‐ZEN‐GCTGGATGGCT‐3IBkFQ‐3′ were used. As a positive amplification control, the housekeeping gene glyceraldehyde‐3‐phosphate dehydrogenase (GAPDH) from *Mus musculus* was used by employing primers GADPH‐Fw 5′‐CAATGTGTCCGTCGTGGATCT‐3′, GADPH‐Rv 5′‐GTCCTCAGTGTAGCCCAAGAT‐3′, and the TaqMan probe GADPH probe 5′‐SUN‐CGTGCCGCC‐ZEN‐TGGAGAAACCTGCC‐3IABkFQ‐3′.^[^ ^107^, ^108^ ^]^ The quantitative RT‐PCR was performed using the kit AgPath‐ID One‐Step RT‐PCR Reagents (Thermo Fisher Scientific) following the manufacturer's recommendation. For quantification purposes, a HA5 standard curve was performed with a synthetic linear DNA that contained one copy of the target HA5 sequence. This DNA was serial diluted in base 10 between 8.3 × 10^7^ copies per reaction until 8.3 copies per reaction. The qPCR was performed as was described previously but replacing the total RNA sample volume for 5 µL of each standard serial dilution. This single standard curve was always performed with the same RT‐qPCR reaction used for all RNA samples analyzed. From this standard curve, the Ct value of each RNA sample was converted in HA5 copies per reaction. Finally, the total copies of HA5 per total RNA extraction were estimated and normalized with respect to the total weight of lungs in milligrams. Thus, each outcome was expressed as gene copies of HA5 per milligram of lung.

### Protein Microarrays

The construction and probing methodology of the influenza protein microarray used for the study had been previously reported.^[^ ^42^, ^109^ ^]^ Briefly, 28 variants of recombinant H5 subtype hemagglutinin, expressed in mammalian or insect cells, were purchased from Sino Biological Inc. and printed as described previously.^[^ ^42^, ^109^ ^]^ The array content and raw data are shown in Table S1 and Figure S3, Supporting Information. For probing, plasma samples were diluted 1:100 in protein array blocking buffer. A His‐tag containing peptide HHHHHHHHGGGG was used to block anti‐polyhistidine antibodies. Plasma samples were incubated with rehydrated arrays in blocking buffer at 4 °C overnight and washed in tris‐buffered saline (TBS) containing 0.05% Tween 20 (T‐TBS) to remove the sera. Bound IgG, IgG1, and IgG2c were detected and visualized using anti‐mouse IgG‐Alexa Fluor 647, IgG1‐Alexa Fluor 647, and IgG2c‐Alexa Fluor 488 (Southern Biotech), respectively. The arrays were incubated with the anti‐mouse detection antibody for 1 h at RT. After washing with T‐TBS to remove non‐specific binding, arrays were air‐dried. The fluorescence intensity of each spot was captured using a Tiny Imager Microarray Imaging System. Spot and background intensities were measured using an annotated grid (.gal) file and captured tiff files quantified using ScanArray express software (Perkin Elmer, Waltham, MA, USA). The background of arrays was subtracted from median spot intensity for each antigen, and the data were normalized.

### Statistical Analyses

Data describing nanoparticle characterization, including hydrodynamic diameter measurements, molecular weights determined by mass spectrometry, and antigen/nanoparticle ratios, are presented as the mean ± standard deviation (S.D.) of at least three independent experiments (*n* ≥ 3), unless otherwise noted. Statistical analysis of immunization data was carried out by using GraphPad Prism. Mouse data are presented as mean ± standard error of the mean (SEM). Antibody and challenge data are gathered from at least five independent individuals (*n* ≥ 5). Lung titer data was gathered from three independent individuals (*n* = 3). Statistical analysis was determined by a one‐way ANOVA over all groups, followed by a Tukey multiple comparisons test, unless otherwise noted. Mantel–Cox log‐rank test was used for survival curve analysis. *P*‐values < 0.05 were considered significant.

## Conflict of Interest

The authors declare no conflict of interest.

## Supporting information



Supporting Information

## Data Availability

The data that support the findings of this study are available from the corresponding author upon reasonable request.
