# Supplementary material for: Co‐Delivery of Multiple Toll‐Like Receptor Agonists and Avian Influenza Hemagglutinin on Protein Nanoparticles Enhances Vaccine Immunogenicity and Efficacy
Source: Adv Healthc Mater. 2025 Feb 9;14(10):2404335. doi: 10.1002/adhm.202404335 (PMC12004444; doi:10.1002/adhm.202404335)
Supplement: Supplementary file 1 — Supporting Information [file ADHM-14-0-s001.pdf]

ADVANCED HEALTHCARE MATERIALS

Supporting Information

for *Adv. Healthcare Mater.*, DOI 10.1002/adhm.202404335

Co-Delivery of Multiple Toll-Like Receptor Agonists and Avian Influenza Hemagglutinin on Protein Nanoparticles Enhances Vaccine Immunogenicity and Efficacy

Aaron Ramirez, Jenny E. Hernandez-Davies, Aarti Jain, Lu Wang, Erwin Strahsburger, D. Huw Davies and Szu-Wen Wang**

Supplementary Information

Co-delivery of multiple toll-like receptor agonists and avian influenza hemagglutinin on protein nanoparticles enhances vaccine immunogenicity and efficacy

Aaron Ramirez[1], Jenny E. Hernandez-Davies[2], Aarti Jain[2], Lu Wang[1], Erwin Strahsburger[2], D. Huw Davies[2,3,*], and Szu-Wen Wang[1,3,4,5,*]

[1]Department of Chemical and Biomolecular Engineering

[2]Vaccine Research and Development Center, Department of Physiology and Biophysics

[3]Institute for Immunology

[4]Department of Biomedical Engineering

[5]Chao Family Comprehensive Cancer Center

University of California

Irvine, California 92697, United States

* corresponding authors:

Szu-Wen Wang, wangsw@uci.edu

Huw Davies, ddavies@uci.edu

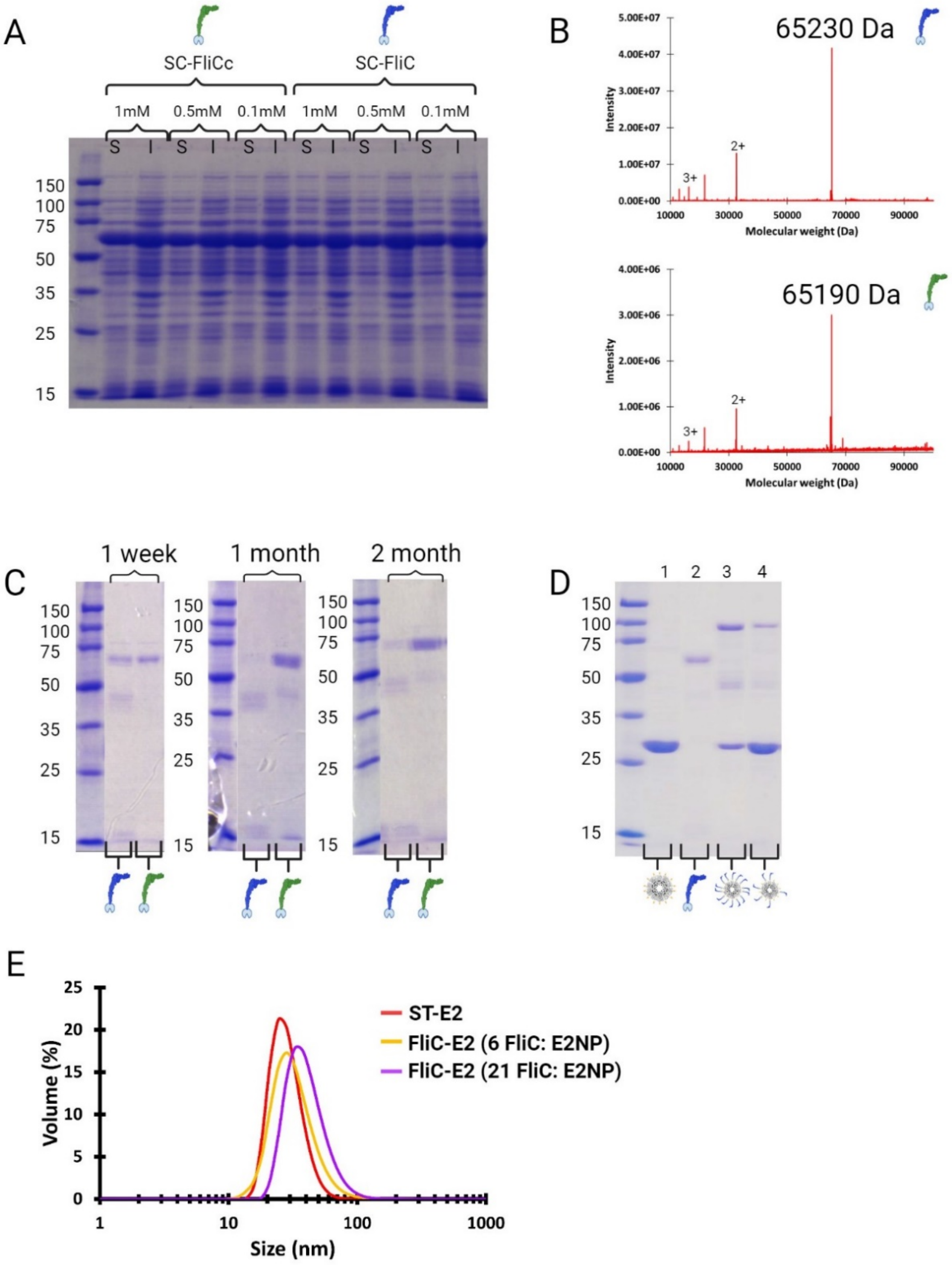

Figure SI-1. Characterization of the purity and stability of SC-FliC and SC-FliCc, and the FliC-E2 nanoparticles. A) Expression of SpyCatcher fused wild type (FliC) and cysteine stabilized (FliCc) flagellin at 37°C induced with 1, 0.5, and 0.1 mM IPTG. $MW_{Flagellin}$ = ~65 kDa. S: soluble; I: insoluble. B) Mass spectrometry of purified SC-FliC (top, blue) and SC-FliCc (bottom, green). C) SDS-PAGE comparing stability of SC-FliCc and SC-FliC after 1 week, 1 month, and 2 months at 4°C. D) SDS-PAGE showing nanoparticles with ~6 and ~21 SC-FliC loaded on the external surface. $MW_{ST\text{-}E2}$ = 30.2 kDa, $MW_{SC\text{-}FliC}$ = 65.2 kDa, $MW_{FliC\text{-}E2}$ = 95.4 kDa. Lanes: 1. ST-E2; 2. SC-FliC; 3. FliC-E2 (6 FliC: E2NP); 4. FliC-E2 (21 FliC: E2NP). E) Hydrodynamic diameters of FliC-E2 NPs with ~6 and ~21 FliC loaded on the external surface.

	A	B	C	D	E	F	G	H
	PBS	ST-E2	SC-H5	H5-E2	H5-FliCc-E2	H5-CpG-E2	H5-FliCc-CpG-E2	SC-H5 + SC-FliCc + CpG + E2

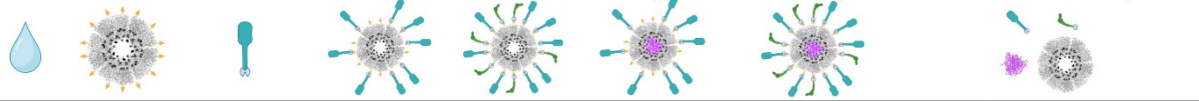

E2	-	3.5 µg (0.12 nmol)	-	3.5 µg (0.12 nmol)	3.5 µg (0.12 nmol)	3.5 µg (0.12 nmol)	3.5 µg (0.12 nmol)	3.5 µg (0.12 nmol)
CpG	-	-	-	-	-	0.2 µg (0.03 nmol)	0.2 µg (0.03 nmol)	0.2 µg (0.03 nmol)
FliCc	-	-	-	-	0.6 µg (0.01 nmol)	-	0.6 µg (0.01 nmol)	0.6 µg (0.01 nmol)
H5	-	-	2 µg (0.03 nmol)	2 µg (0.03 nmol)	2 µg (0.03 nmol)	2 µg (0.03 nmol)	2 µg (0.03 nmol)	2 µg (0.03 nmol)

Figure SI-2. Table describing each formulation and its individual components in mass and mole amounts. 3.5 µg of E2 is equivalent to 0.12 nmol of E2. 0.2 µg of CpG is equivalent to 0.03 nmol of CpG. 0.6 µg of FliCc is equivalent to 0.01 nmol of FliCc. 2 µg of H5 is equivalent to 0.03 nmol of H5.

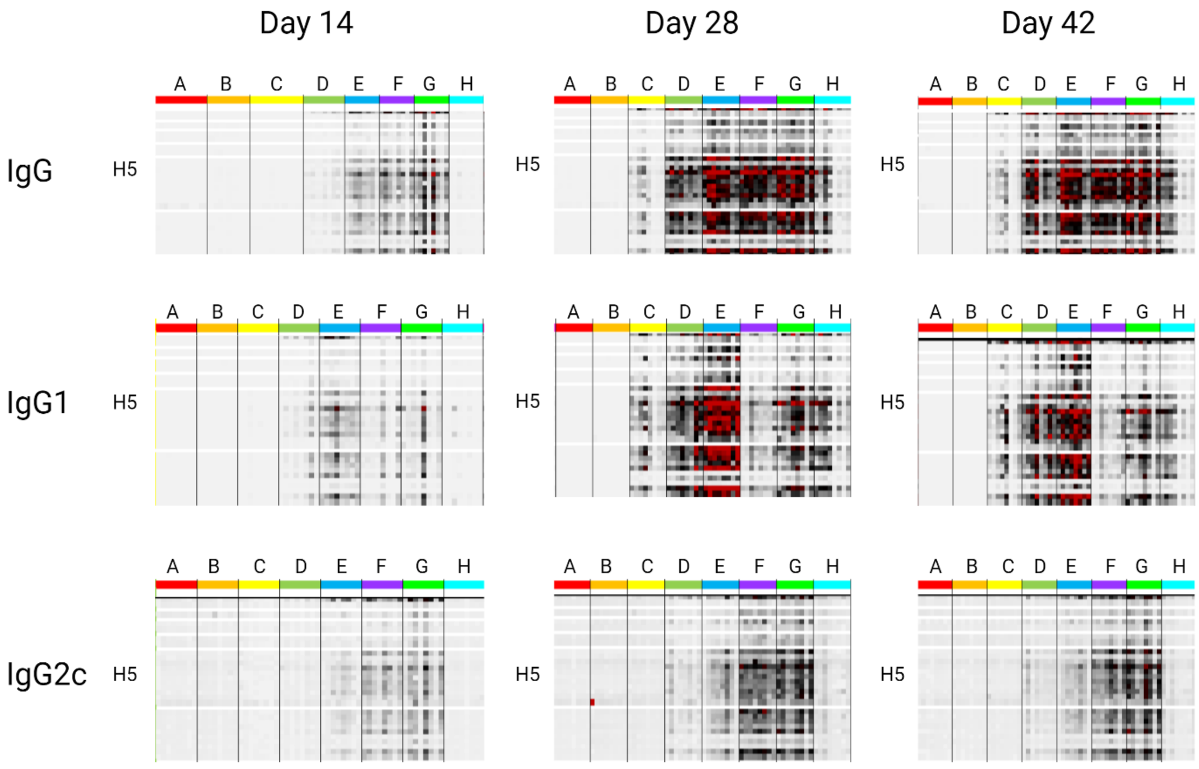

Figure SI-3. Antibody arrays from sera of immunized mice. Eight groups of 8 mice (Groups A to H) were administered different formulations on day 0 and boosted on day 14. IgG, IgG1, and IgG2c signal intensities were determined at different time points post-prime against H5 variants displayed on protein microarrays. Microarray data was compiled into heat map compilations with columns representing immunized mice groups and rows representing individual H5 variants (i.e., 28 variants of H5 were analyzed) (red = high amount of binding, white = low amount of binding). Group A: PBS, Group B: ST-E2, Group C: SC-H5, Group D: H5-E2, Group E: H5-FliCc-E2, Group F: H5-CpG-E2, Group G: H5-FliCc-CpG-E2, Group H: SC-H5 + SC-FliCc + CpG + E2.

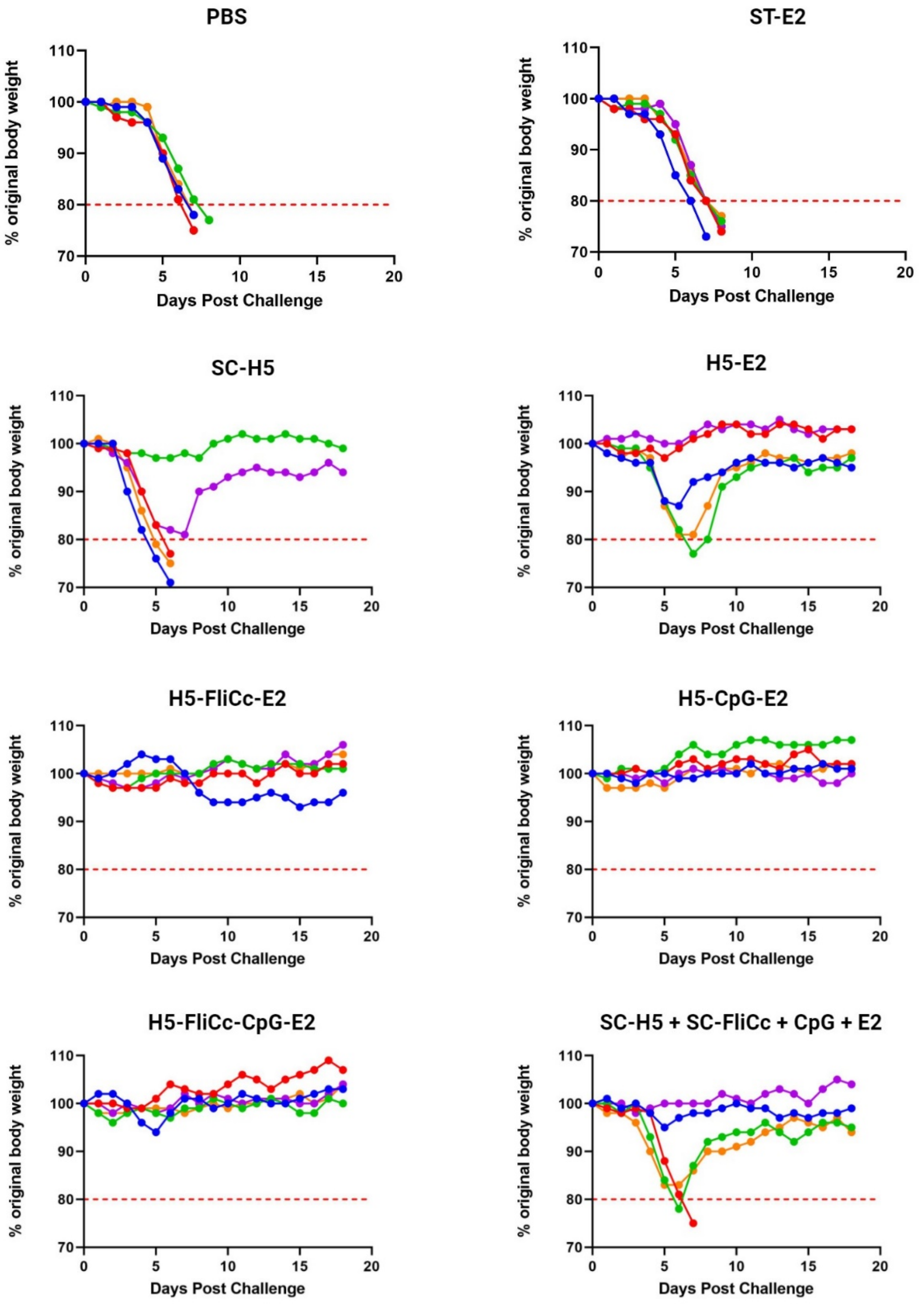

Figure SI-4. Individual weight curves of mice during H5N1 challenge. Mice weights and physical state were monitored over 18 days after H5N1 virus inoculation. Each group had 5 mice. One mouse from the PBS group was evaluated to be an outlier using Grubb's outlier test comparing maximum weight loss between mice. The red dotted line indicates 20% weight loss.

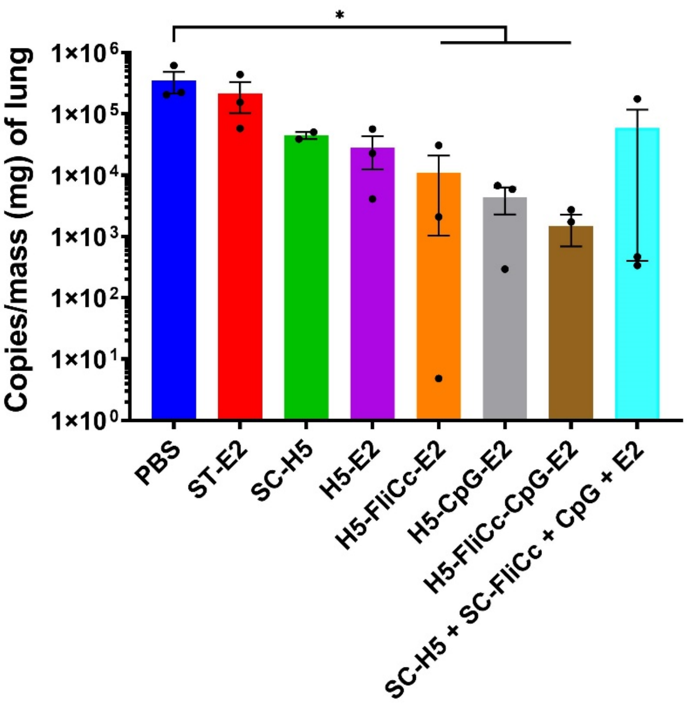

Figure SI-5. Viral loads in lungs four days post-challenge. Influenza hemagglutinin H5 gene quantification by qPCR, represented as gene copies of hemagglutinin H5 normalized to the weight of the lungs. Each group had 3 mice. (Data of one sample from the SC-H5 group could not be acquired. Data of one sample from the H5-FliCc-CpG-E2 group was between 0-1 copy/mg of lung.). Statistical significance was determined by one-way ANOVA followed by a Tukey multiple comparisons test. *$p < 0.05$.

Variant #	Sino Biological Catalog #	Influenza Strain/Control ID	Subtype	Influenza Type	Expression System	Protein Sequence
1	11710-V08H	A/Cambodia/R0405050/2007	H5N1	A	HEK293 Cells	Met1-Gln531
2	11713-V08H	A/HongKong/213/2003	H5N1	A	HEK293 Cells	Met1-Gln531,28Ser/Trp
3	40088-V08H	A/chicken/Yamaguchi/7/2004	H5N1	A	HEK293 Cells	Met1-Gln530
4	11697-V08H	A/Egypt/2321NAMRU3/2007	H5N1	A	HEK293 Cells	Met1-Gln531
5	11062-V08H1	A/Vietnam/1194/2004	H5N1	A	HEK293 Cells	Met1-Gln531
6	11689-V08H	A/HongKong/483/1997	H5N1	A	HEK293 Cells	Met1-Gln531
7	11712-V08H	A/chicken/India/NIV33487/2006	H5N1	A	HEK293 Cells	Met1-Gln531
8	11690-V08H	A/goose/Guiyang/337/2006	H5N1	A	HEK293 Cells	Met1-Gln531
9	40001-V08H	A/duck/HongKong/P46/1997	H5N1	A	HEK293 Cells	Met1-Gln539
10	11709-V08H	A/whooperswan/Mongolia/244/2005	H5N1	A	HEK293 Cells	Met1-Gln531
11	40026-V08H	A/Cambodia/S1211394/2008	H5N1	A	HEK293 Cells	Met1-Gly341
12	11698-V08H	A/duck/Hunan/795/2002	H5N1	A	HEK293 Cells	Met1-Gln531
13	11694-V08H	A/japanesewhiteeye/HongKong/1038/2006	H5N1	A	HEK293 Cells	Met1-Gln530
14	40004-V08H	A/Xinjiang/1/2006	H5N1	A	HEK293 Cells	Met1-Gln531
15	11700-V08H	A/commonmagpie/HongKong/2256/2006	H5N1	A	HEK293 Cells	Met1-Glu530
16	11702-V08H	A/Egypt/N05056/2009	H5N1	A	HEK293 Cells	Met1-Gln530
17	40044-V08H	A/commonmagpie/HongKong/5052/2007	H5N1	A	HEK293 Cells	Met1-Gln530
18	40117-V08B	A/barheadedgoose/Qinghai/1A/2005	H5N1	A	Baculovirus-Insect Cells	Met1-Gln527
19	40015-V08H	A/Hubei/1/2010	H5N1	A	HEK293 Cells	Met1-Gln530
20	40024-V08B	A/goose/Guangdong/1/1996	H5N1	A	Baculovirus-Insect Cells	Met1-Gln531
21	40022-V08H	A/Vietnam/UT31413II/2008	H5N1	A	HEK293 Cells	Met1-Gln530
22	11062-V08B-B	H5N1 (A/Vietnam/1194/2004) Hemagglutinin / HA Protein (His Tag), Biotinylated	H5N1	A	Baculovirus-Insect Cells	Met1-Gln531
23	11048-V08H4	A/Anhui/1/2005	H5N1	A	HEK293 Cells	Met1-Gln530
24	40372-V08B	A/chicken/Jilin/9/2004	H5N1	A	Baculovirus-Insect Cells	Met1-Gln531
25	40160-V08B	A/barnswallow/HongKong/D101161/2010	H5N1	A	Baculovirus-Insect Cells	Met1-Gln531
26	40158-V08B	A/chicken/Vietnam/NCVD016/2008	H5N1	A	Baculovirus-Insect Cells	Met1-Gln532
27	11060-V08B1	H5N1 (A/Indonesia/5/2005) Hemagglutinin / HA-specific B cell probe (His Tag)	H5N1	A	Baculovirus-Insect Cells	Met1-Ile519
28	11060-V08B1	H5N1 (A/Indonesia/5/2005) Hemagglutinin / HA-specific B cell probe (His Tag)	H5N1	A	Baculovirus-Insect Cells	Met1-Ile519

Table SI-1. H5 variants printed on the protein microarray used for IgG, IgG1, and IgG2c antibody profiling. H5 subtype descriptions: variant number, Sino Biological catalog number, influenza strain, subtype, and type, expression system, protein sequence.

Abbreviation	Description: DNA (top) and protein (bottom) sequences
SC-FliCc (N-terminus truncated SpyCatcher fused with cysteine-modified flagellin)	ATGTCGTACTACCATCACCATCACCATCACGATTACGACATCCCAA<br>CGACCGAAAACCTGTATTTTCAGGGCGATAGTGCTACCCATATTA<br>AATTCTCAAAACGTGATGAGGACGGCAAAGAGTTAGCTGGTGCA<br>ACTATGGAGTTGCGTGATTCATCTGGTAAAACTATTAGTACATGGA<br>TTTCAGATGGACAAGTGAAAGATTTCTACCTGTATCCAGGAAAATA<br>TACATTTGTCGAAACCGCAGCACCAGACGGTTATGAGGTAGCAA<br>CTGCTATTACCTTTACAGTTAATGAGCAAGGTCAGGTTACTGTAAA<br>TGGCAAAGCAACTAAAGGTGACGCTCATATTGCTAGCATGGGATC<br>AGGGGGATCAGGTGGCAGCGGAATACAAGTGATTAATACAAACA<br>GCCTGTCGCTGTTGACCCAGAATAACCTGAACAAATCCCAGTCC<br>GCTCTGGGCACCGCTATCGAGCGTCTGTCTTCCGGTTGCCGTAT<br>CAACAGCGCGAAAGACGATGCGGCAGGTCAGGCGATTGCTAAC<br>CGTTTTACCGCGAACATCAAAGGTCTGACTCAGGCTTCCCGTAA<br>CGCTAACGACGGTATCTCCATTGCGCAGACCACTGAAGGCGCGC<br>TGAACGAAATCAACAACAACCTGCAGCGTGTGCGTGAACTGGCG<br>GTTCAGTCTGCTAACAGCACCAACTCCCAGTCTGACCTCGACTC<br>CATCCAGGCTGAAATCACCCAGCGCCTGAACGAAATCGACCGTG<br>TATCCGGCCAGACTCAGTTCAACGGCGTGAAAGTCCTGGCGCA<br>GGACAACACCCTGACCATCCAGGTTGGTGCCAACGACGGTGAA<br>ACTATCGATATCGATCTGAAGCAGATCAACTCTCAGACCCTGGGT<br>CTGGATACGCTGAATGTGCAACAAAAATATAAGGTCAGCGATACG<br>GCTGCAACTGTTACAGGATATGCCGATACTACGATTGCTTTAGAC<br>AATAGTACTTTTAAAGCCTCGGCTACTGGTCTTGGTGGTACTGAC<br>CAGAAAATTGATGGCGATTTAAAATTTGATGATACGACTGGAAAAT<br>ATTACGCCAAAGTTACCGTTACGGGGGGAACTGGTAAAGATGGC<br>TATTATGAAGTTTCCGTTGATAAGACGAACGGTGAGGTGACTCTT<br>GCTGGCGGTGCGACTTCCCCGCTTACAGGTGGACTACCTGCGA<br>CAGCAACTGAGGATGTGAAAAATGTACAAGTTGCAAATGCTGATT<br>TGACAGAGGCTAAAGCCGCATTGACAGCAGCAGGTGTTACCGGC<br>ACAGCATCTGTTGTTAAGATCTCTTATACTGATAATAACGGTAAAA<br>CTATTGATGGTGGTTTAGCAGTTAAGGTAGGCGATGATTACTATTC<br>TGCAACTCAAAATAAAGATGGTTCCATAAGTATTAATACTACGAAAT<br>ACACTGCAGATGACGGTACATCCAAAACTGCACTAAACAAACTGG<br>GTGGCGCAGACGGCAAAACCGAAGTTGTTTCTATTGGTGGTAAA<br>ACTTACGCTGCAAGTAAAGCCGAAGGTCACAACTTTAAAGCACA<br>GCCTGATCTGGCGGAAGCGGCTGCTACAACCACCGAAAACCCG<br>CTGCAGAAAATTGATGCTGCTTTGGCACAGGTTGACACGTTACGT<br>TCTGACCTGGGTGCGGTACAGAACCGTTTCAACTCCGCTATTAC<br>CAACCTGGGCAACACCGTAAACAACCTGACTTCTGCCCGTAGCC<br>GTATCTGTGATTCCGACTACGCGACCGAAGTTTCCAACATCTCTC<br>GCGCGCAGATTCTGCAGCAGGCCGGTACCTCCGTTCTGGCGCA<br>GGCGAACCAGGTTCCGCAAAACGTCCTCTCTTTACTGCGTTAA<br><br>MSYYHHHHHHDYDIPTTENLYFQGDSATHIKFSKRDEDGKELAGAT<br>MELRDSSGKTISTWISDGQVKDFYLYPGKYTFVETAAPDGYEVATAI<br>TFTVNEQGQVTVNGKATKGDAHIASMGSGGSGGSGIQVINTNSLSL<br>LTQNNLNKSQSALGTAIERLSSGCRINSAKDDAAGQAIANRFTANIK

	GLTQASRNANDGISIAQTTEGALNEINNNLQRVRELAVQSANSTNSQ<br>SDLDSIQAEITQRLNEIDRVSGQTQFNGVKVLAQDNTLTIQVGANDG<br>ETIDIDLKQINSQTLGLDTLNVQQKYKVSDTAATVTGYADTTIALDNS<br>TFKASATGLGGTDQKIDGDLKFDDTTGKYYAKVTVTGGTGKDGYYE<br>VSVDKTNGEVTLAGGATSPLTGGLPATATEDVKNVQVANADLTEAK<br>AALTAAGVTGTASVVKISYTDNNGKTIDGGLAVKVGDDYYSATQNKD<br>GSISINTTKYTADDGTSKTALNKLGGADGKTEVVSIGGKTYAASKAE<br>GHNFKAQPDLAEAAATTTENPLQKIDAALAQVDTLRSDLGAVQNRF<br>NSAITNLGNTVNNLTSARSRICDSDYATEVSNISRAQILQQAGTSVLA<br>QANQVPQNVLSLLR*
SC-FliC (N-terminus truncated SpyCatcher fused with wild-type flagellin)	ATGTCGTACTACCATCACCATCACCATCACGATTACGACATCCCAA<br>CGACCGAAAACCTGTATTTTCAGGGCGATAGTGCTACCCATATTA<br>AATTCTCAAAACGTGATGAGGACGGCAAAGAGTTAGCTGGTGCA<br>ACTATGGAGTTGCGTGATTCATCTGGTAAAACTATTAGTACATGGA<br>TTTCAGATGGACAAGTGAAAGATTTCTACCTGTATCCAGGAAAATA<br>TACATTTGTCGAAACCGCAGCACCAGACGGTTATGAGGTAGCAA<br>CTGCTATTACCTTTACAGTTAATGAGCAAGGTCAGGTTACTGTAAA<br>TGGCAAAGCAACTAAAGGTGACGCTCATATTGCTAGCATGGGATC<br>AGGGGGATCAGGTGGCAGCGGAGCACAAGTGATTAATACAAACA<br>GCCTGTCGCTGTTGACCCAGAATAACCTGAACAAATCCCAGTCC<br>GCTCTGGGCACCGCTATCGAGCGTCTGTCTTCCGGTCTGCGTAT<br>CAACAGCGCGAAAGACGATGCGGCAGGTCAGGCGATTGCTAAC<br>CGTTTTACCGCGAACATCAAAGGTCTGACTCAGGCTTCCCGTAA<br>CGCTAACGACGGTATCTCCATTGCGCAGACCACTGAAGGCGCGC<br>TGAACGAAATCAACAACAACCTGCAGCGTGTGCGTGAACTGGCG<br>GTTCAGTCTGCTAACAGCACCAACTCCCAGTCTGACCTCGACTC<br>CATCCAGGCTGAAATCACCCAGCGCCTGAACGAAATCGACCGTG<br>TATCCGGCCAGACTCAGTTCAACGGCGTGAAAGTCCTGGCGCA<br>GGACAACACCCTGACCATCCAGGTTGGTGCCAACGACGGTGAA<br>ACTATCGATATCGATCTGAAGCAGATCAACTCTCAGACCCTGGGT<br>CTGGATACGCTGAATGTGCAACAAAAATATAAGGTCAGCGATACG<br>GCTGCAACTGTTACAGGATATGCCGATACTACGATTGCTTTAGAC<br>AATAGTACTTTTAAAGCCTCGGCTACTGGTCTTGGTGGTACTGAC<br>CAGAAAATTGATGGCGATTTAAAATTTGATGATACGACTGGAAAAT<br>ATTACGCCAAAGTTACCGTTACGGGGGGAACTGGTAAAGATGGC<br>TATTATGAAGTTTCCGTTGATAAGACGAACGGTGAGGTGACTCTT<br>GCTGGCGGTGCGACTTCCCCGCTTACAGGTGGACTACCTGCGA<br>CAGCAACTGAGGATGTGAAAAATGTACAAGTTGCAAATGCTGATT<br>TGACAGAGGCTAAAGCCGCATTGACAGCAGCAGGTGTTACCGGC<br>ACAGCATCTGTTGTTAAGATGTCTTATACTGATAATAACGGTAAAA<br>CTATTGATGGTGGTTTAGCAGTTAAGGTAGGCGATGATTACTATTC<br>TGCAACTCAAAATAAAGATGGTTCCATAAGTATTAATACTACGAAAT<br>ACACTGCAGATGACGGTACATCCAAAACTGCACTAAACAAACTGG<br>GTGGCGCAGACGGCAAAACCGAAGTTGTTTCTATTGGTGGTAAA<br>ACTTACGCTGCAAGTAAAGCCGAAGGTCACAACTTTAAAGCACA<br>GCCTGATCTGGCGGAAGCGGCTGCTACAACCACCGAAAACCCG<br>CTGCAGAAAATTGATGCTGCTTTGGCACAGGTTGACACGTTACGT<br>TCTGACCTGGGTGCGGTACAGAACCGTTTCAACTCCGCTATTAC<br>CAACCTGGGCAACACCGTAAACAACCTGACTTCTGCCCGTAGCC<br>GTATCGAAGATTCCGACTACGCGACCGAAGTTTCCAACATGTCTC

	GCGCGCAGATTCTGCAGCAGGCCGGTACCTCCGTTCTGGCGCA GGCGAACCAGGTTCCGCAAAACGTCCTCTCTTTACTGCGTTAA<br><br>MSYYHHHHHHDYDIPTTENLYFQGDSATHIKFSKRDEDGKELAGAT MELRDSSGKTISTWISDGQVKDFYLYPGKYTFVETAAPDGYEVATAI TFTVNEQGQVTVNGKATKGDAHIASMGSGGSGGSGAQVINTNSLS LLTQNNLNKSQSALGTAIERLSSGLRINSAKDDAAGQAIANRFTANIK GLTQASRNANDGISIAQTTEGALNEINNNLQRVRELAVQSANSTNSQ SDLDSIQAEITQRLNEIDRVSGQTQFNGVKVLAQDNTLTIQVGANDG ETIDIDLKQINSQTLGLDTLNVQQKYKVSDTAATVTGYADTTIALDNS TFKASATGLGGTDQKIDGDLKFDDTTGKYYAKVTVTGGTGKDGYYE VSVDKTNGEVTLAGGATSPLTGGLPATATEDVKNVQVANADLTEAK AALTAAGVTGTASVVKMSYTDNNGKTIDGGLAVKVGDDYYSATQNK DGSISINTTKYTADDGTSKTALNKLGGADGKTEVVSIGGKTYAASKA EGHNFKAQPDLAEAAATTTENPLQKIDAALAQVDTLRSDLGAVQNR FNSAITNLGNTVNNLTSARSRIEDSDYATEVSNMSRAQILQQAGTSV LAQANQVPQNVLSLLR*
H5 (Transmembrane domain truncated H5 hemagglutinin from A/Vietnam/1194/2004)	GATCAGATTTGCATTGGTTACCATGCAAACAACTCGACAGAGCAG GTTGACACAATAATGGAAAAGAACGTTACTGTTACACATGCCCAA GACATACTGGAAAAGACACACAATGGGAAGCTCTGCGATCTAGAT GGAGTGAAGCCTCTAATTTTGAGAGATTGTAGTGTAGCTGGATGG CTCCTCGGAAACCCAATGTGTGACGAATTCATCAATGTGCCGGA ATGGTCTTACATAGTGGAGAAGGCCAATCCAGTCAATGACCTCTG TTACCCAGGGGATTTCAATGACTATGAAGAATTGAAACACCTATTG AGCAGAATAAACCATTTTGAGAAAATTCAGATCATCCCCAAAAGTT CTTGGTCCAGTCATGAAGCCTCATTGGGGGTGAGCTCAGCATGT CCATACCAGGGAAAGTCCTCCTTTTTCAGAAATGTGGTATGGCTT ATCAAAAAGAACAGTACATACCCAACAATAAAGAGGAGCTACAATA ATACCAACCAAGAAGATCTTTTGGTACTGTGGGGGATTCACCATC CTAATGATGCGGCAGAGCAGACAAAGCTCTATCAAAACCCAACC ACCTATATTTCCGTTGGGACATCAACACTAAACCAGAGATTGGTA CCAAGAATAGCTACTAGATCCAAAGTAAACGGGCAAAGTGGAAG GATGGAGTTCTTCTGGACAATTTTAAAACCGAATGATGCAATCAA CTTCGAGAGTAATGGAAATTTCATTGCTCCAGAATATGCATACAAA ATTGTCAAGAAAGGGGACTCAACAATTATGAAAAGTGAATTGGAA TATGGTAACTGCAACACCAAGTGTCAAACTCCAATGGGGGCGATA AACTCTAGCATGCCATTCCACAATATACACCCTCTCACCATCGGG GAATGCCCCAAATATGTGAAATCAAACAGATTAGTCCTTGCGACT GGGCTCAGAAATAGCCCTCAACGAGAGACGCGAGGATTATTTGG AGCTATAGCAGGTTTTATAGAGGGAGGATGGCAGGGAATGGTAG ATGGTTGGTATGGGTACCACCATAGCAACGAGCAGGGGAGTGGG TACGCTGCAGACAAAGAATCCACTCAAAAGGCAATAGATGGAGT CACCAATAAGGTCAACTCGATTATTGACAAAATGAACACTCAGTTT GAGGCCGTTGGAAGGGAATTTAACAACTTAGAAAGGAGAATAGA GAATTTAAACAAGAAGATGGAAGACGGGTTCCTAGATGTCTGGAC TTATAATGCTGAACTTCTAGTTCTCATGGAAAACGAGAGAACTCTA GACTTTCATGACTCAAATGTCAAGAACCTTTACGACAAGGTCCGA CTACAGCTTAGGGATAATGCAAAGGAGCTGGGTAACGGTTGTTTC GAGTTCTATCATAAATGTGATAATGAATGTATGGAAAGTGTAAGAA

	ACGGAACGTATGACTACCCGCAGTATTCAGAAGAAGCAAGACTAA AAAGAGAGGAAATAAGTGGAGTAAAATTGGAATCAATAGGAATTTA CCAAATATAA<br><br>DQICIGYHANNSTEQVDTIMEKNVTVTHAQDILEKTHNGKLCDLDGV KPLILRDCSVAGWLLGNPMCDEFINVPEWSYIVEKANPVNDLCYPG DFNDYEELKHLLSRINHFEKIQIIPKSSWSSHEASLGVSSACPYQGK SSFFRNVVWLIKKNSTYPTIKRSYNNTNQEDLLVLWGIHHPNDAAEQ TKLYQNPTTYISVGTSTLNQRLVPRIATRSKVNGQSGRMEFFWTILK PNDAINFESNGNFIAPEYAYKIVKKGDSTIMKSELEYGNCNTKCQTP MGAINSSMPFHNIHPLTIGECPKYVKSNRLVLATGLRNSPQRETRGL FGAIAGFIEGGWQGMVDGWYGYHHSNEQGSGYAADKESTQKAID GVTNKVNSIIDKMNTQFEAVGREFNNLERRIENLNKKMEDGFLDVW TYNAELLVLMENERTLDFHDSNVKNLYDKVRLQLRDNAKELGNGCF EFYHKCDNECMESVRNGTYDYPQYSEEARLKREEISGVKLESIGIY QI*
SC-H5 (N-terminus truncated SpyCatcher fused with H5 hemagglutinin)	ATGTACAGGATGCAACTCCTGTCTTGCATTGCACTAAGTCTTGCA CTTGTCACAAACAGTCATCATCACCATCATCACCATCACCATTCAG GAGGAGGATCAGGAGGAGGAATCGAGGGAAGGGATAGTGCTAC CCATATTAAATTCTCAAAACGTGATGAGGACGGCAAAGAGTTAGC TGGTGCAACTATGGAGTTGCGTGATTCATCTGGTAAAACTATTAGT ACATGGATTTCAGATGGACAAGTGAAAGATTTCTACCTGTATCCA GGAAAATATACATTTGTCGAAACCGCAGCACCAGACGGTTATGAG GTAGCAACTGCTATTACCTTTACAGTTAATGAGCAAGGTCAGGTTA CTGTAAATGGCAAAGCAACTAAAGGTGACGCTCATATTGCTAGCG GTTCAGGAACAGCAGGTGGTGGGTCAGGTTCCGATCAGATTTGC ATTGGTTACCATGCAAACAACTCGACAGAGCAGGTTGACACAATA ATGGAAAAGAACGTTACTGTTACACATGCCCAAGACATACTGGAA AAGACACACAATGGGAAGCTCTGCGATCTAGATGGAGTGAAGCC TCTAATTTTGAGAGATTGTAGTGTAGCTGGATGGCTCCTCGGAAA CCCAATGTGTGACGAATTCATCAATGTGCCGGAATGGTCTTACAT AGTGGAGAAGGCCAATCCAGTCAATGACCTCTGTTACCCAGGGG ATTTCAATGACTATGAAGAATTGAAACACCTATTGAGCAGAATAAA CCATTTTGAGAAAATTCAGATCATCCCCAAAAGTTCTTGGTCCAG TCATGAAGCCTCATTGGGGGTGAGCTCAGCATGTCCATACCAGG GAAAGTCCTCCTTTTTCAGAAATGTGGTATGGCTTATCAAAAAGA ACAGTACATACCCAACAATAAAGAGGAGCTACAATAATACCAACCA AGAAGATCTTTTGGTACTGTGGGGGATTCACCATCCTAATGATGC GGCAGAGCAGACAAAGCTCTATCAAAACCCAACCACCTATATTTC CGTTGGGACATCAACACTAAACCAGAGATTGGTACCAAGAATAGC TACTAGATCCAAAGTAAACGGGCAAAGTGGAAGGATGGAGTTCTT CTGGACAATTTTAAAACCGAATGATGCAATCAACTTCGAGAGTAAT GGAAATTTCATTGCTCCAGAATATGCATACAAAATTGTCAAGAAAG GGGACTCAACAATTATGAAAAGTGAATTGGAATATGGTAACTGCA ACACCAAGTGTCAAACTCCAATGGGGGCGATAAACTCTAGCATG CCATTCCACAATATACACCCTCTCACCATCGGGGAATGCCCCAAA TATGTGAAATCAAACAGATTAGTCCTTGCGACTGGGCTCAGAAAT AGCCCTCAACGAGAGACGCGAGGATTATTTGGAGCTATAGCAGG TTTTATAGAGGGAGGATGGCAGGGAATGGTAGATGGTTGGTATGG

	GTACCACCATAGCAACGAGCAGGGGAGTGGGTACGCTGCAGAC<br>AAAGAATCCACTCAAAAGGCAATAGATGGAGTCACCAATAAGGTC<br>AACTCGATTATTGACAAAATGAACACTCAGTTTGAGGCCGTTGGA<br>AGGGAATTTAACAACTTAGAAAGGAGAATAGAGAATTTAAACAAG<br>AAGATGGAAGACGGGTTCCTAGATGTCTGGACTTATAATGCTGAA<br>CTTCTAGTTCTCATGGAAAACGAGAGAACTCTAGACTTTCATGAC<br>TCAAATGTCAAGAACCTTTACGACAAGGTCCGACTACAGCTTAGG<br>GATAATGCAAAGGAGCTGGGTAACGGTTGTTTCGAGTTCTATCAT<br>AAATGTGATAATGAATGTATGGAAAGTGTAAGAAACGGAACGTATG<br>ACTACCCGCAGTATTCAGAAGAAGCAAGACTAAAAAGAGAGGAA<br>ATAAGTGGAGTAAAATTGGAATCAATAGGAATTTACCAAATATAA<br><br>MYRMQLLSCIALSLALVTNSHHHHHHHHHHSGGGSGGGIEGRDSAT<br>HIKFSKRDEDGKELAGATMELRDSSGKTISTWISDGQVKDFYLYPG<br>KYTFVETAAPDGYEVATAITFTVNEQGQVTVNGKATKGDAHIASGSG<br>TAGGGSGSDQICIGYHANNSTEQVDTIMEKNVTVTHAQDILEKTHN<br>GKLCDLDGVKPLILRDCSVAGWLLGNPMCDEFINVPEWSYIVEKAN<br>PVNDLCYPGDFNDYEELKHLLSRINHFEKIQIIPKSSWSSHEASLGV<br>SSACPYQGKSSFFRNVVWLIKKNSTYPTIKRSYNNTNQEDLLVLWGI<br>HHPNDAAEQTKLYQNPTTYISVGTSTLNQRLVPRIATRSKVNGQSG<br>RMEFFWTILKPNDAINFESNGNFIAPEYAYKIVKKGDSTIMKSELEYG<br>NCNTKCQTPMGAINSSMPFHNIHPLTIGECPKYVKSNRLVLATGLRN<br>SPQRETRGLFGAIAGFIEGGWQGMVDGWYGYHHSNEQGSGYAAD<br>KESTQKAIDGVTNKVNSIIDKMNTQFEAVGREFNNLERRIENLNKKM<br>EDGFLDVWTYNAELLVLMENERTLDFHDSNVKNLYDKVRLQLRDNA<br>KELGNGCFEFYHKCDNECMESVRNGTYDYPQYSEEARLKREEISG<br>VKLESIGIYQI*

Table SI-2. DNA and protein sequences of antigen mutants.
